# Supplementary material for: A novel bacterial effector protein mediates ER-LD membrane contacts to regulate host lipid droplets
Source: EMBO Rep. 2024 Sep 27;25(12):11. doi: 10.1038/s44319-024-00266-8 (PMC11624262; doi:10.1038/s44319-024-00266-8)
Supplement: Supplementary file 2 — Source data Fig. 3 [file 44319_2024_266_MOESM2_ESM.zip › EMBOR-2024-59287-SourceDataForFigure3C,3G,3H/3C/READ ME_Data processing and presentation.docx]

Data processing and presentation:

Cultures grown on agar plate was imaged using mobile camera. Area containing five culture spots were cropped for data presentation.
