# Supplementary material for: A novel bacterial effector protein mediates ER-LD membrane contacts to regulate host lipid droplets
Source: EMBO Rep. 2024 Sep 27;25(12):11. doi: 10.1038/s44319-024-00266-8 (PMC11624262; doi:10.1038/s44319-024-00266-8)
Supplement: Supplementary file 2 — Source data Fig. 3 [file 44319_2024_266_MOESM2_ESM.zip › EMBOR-2024-59287-SourceDataForFigure3C,3G,3H/3G/READ ME_Data processing and presentation.docx]

Data processing and presentation:

Input and pulldown (Immunoprecipitation or IP) fractions were run and developed as separate blots.

Each blot was probed with anti-GFP, anti-VAPB and anti-MOSPD2. After development of each signal, the blot was reused post washing with stripping buffer (Thermo Scientific, Catalog number -21059).

Correct signal was identified and selected based on the published and predicted molecular weights for each protein.

Images were flipped from right to left where needed for better presentation of the data.
