# Supplementary figures and images for: A novel bacterial effector protein mediates ER-LD membrane contacts to regulate host lipid droplets
Source: EMBO Rep. 2024 Sep 27;25(12):11. doi: 10.1038/s44319-024-00266-8 (PMC11624262; doi:10.1038/s44319-024-00266-8)

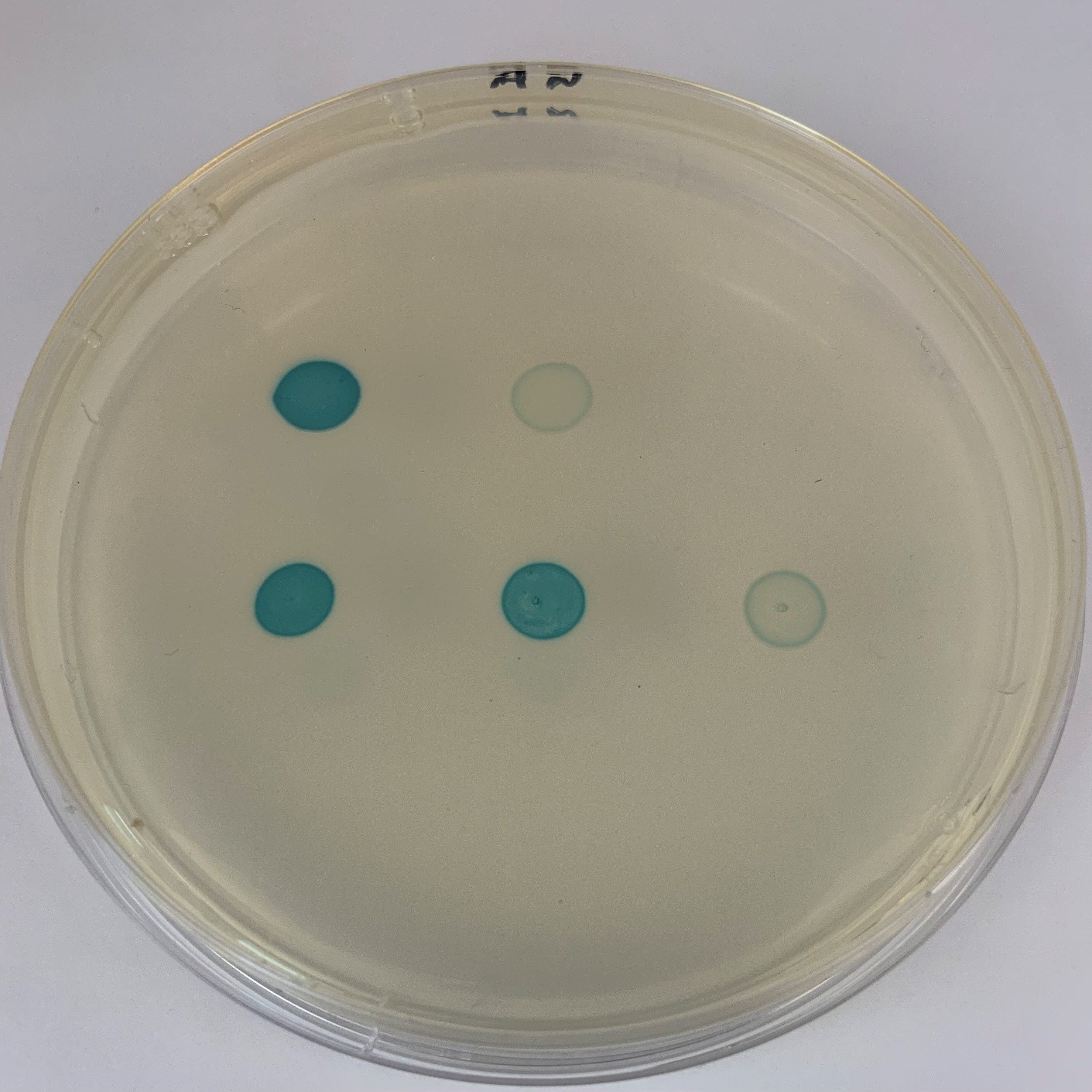

Supplement: Supplementary file 2 — Source data Fig. 3 [file 44319_2024_266_MOESM2_ESM.zip › EMBOR-2024-59287-SourceDataForFigure3C,3G,3H/3C/BACTH assay spotting.jpg]

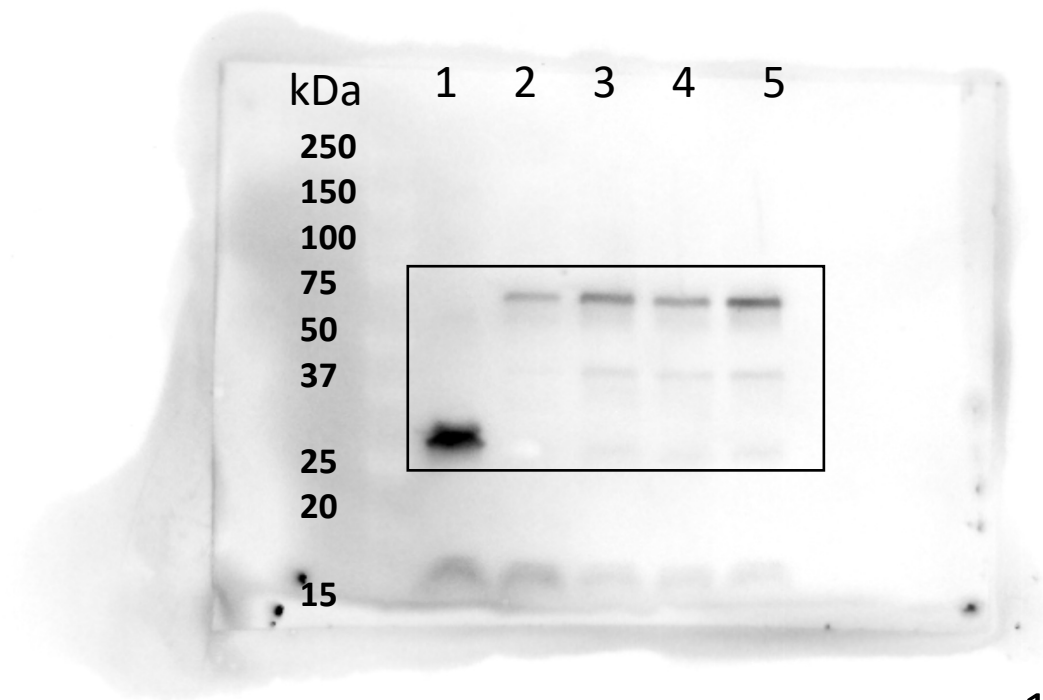

Anti-GFP

- 1 GFP
- 2 *CbEPF1*-wt-GFP
- 3 *CbEPF1*-F1mt-GFP
- 4 *CbEPF1*-F2mt-GFP
- 5 *CbEPF1*-F3mt-GFP

Supplement: Supplementary file 2 — Source data Fig. 3 [file 44319_2024_266_MOESM2_ESM.zip › EMBOR-2024-59287-SourceDataForFigure3C,3G,3H/3G/Blot_IP_anti-GFP-annotated.pdf]

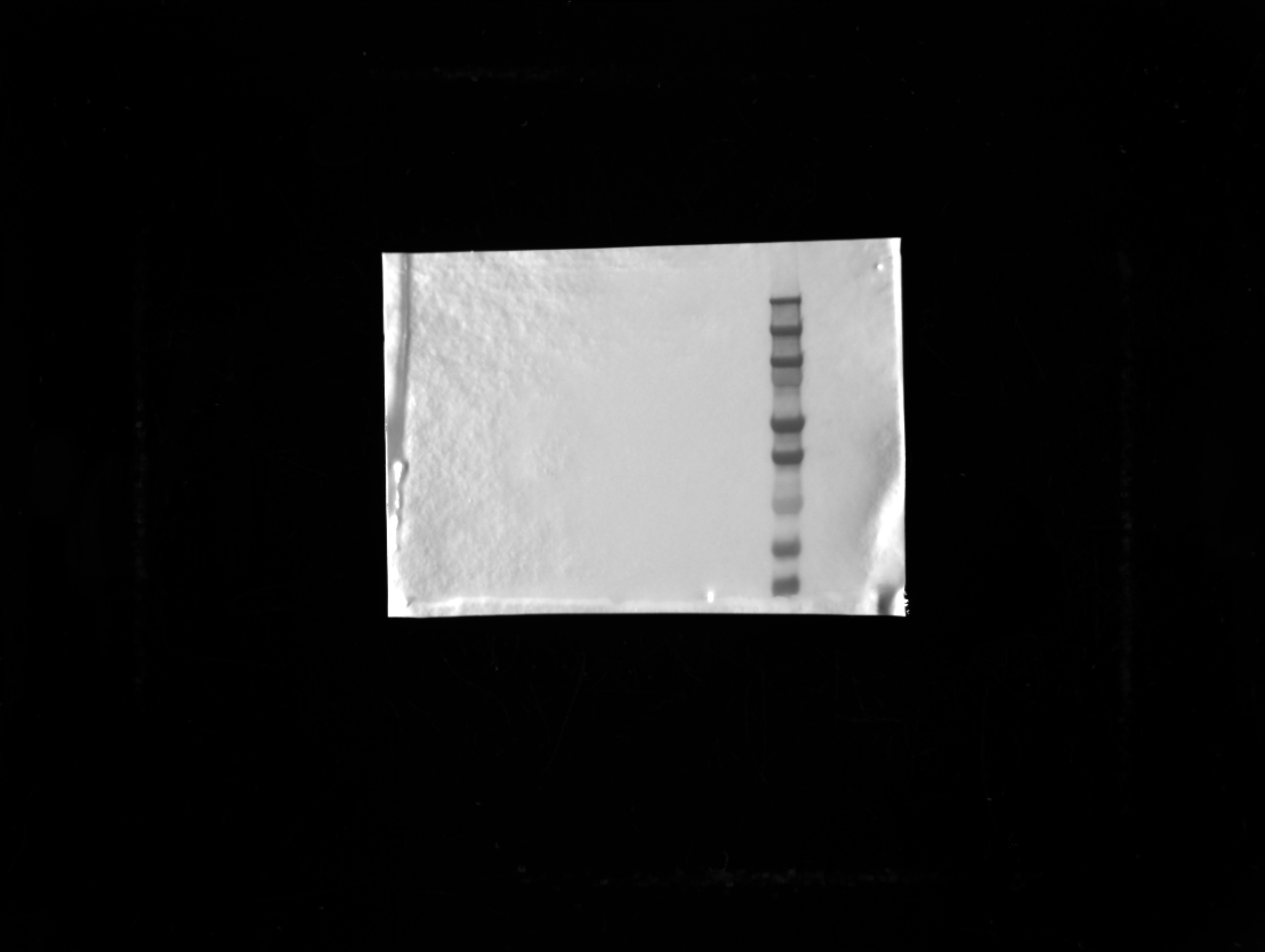

Supplement: Supplementary file 2 — Source data Fig. 3 [file 44319_2024_266_MOESM2_ESM.zip › EMBOR-2024-59287-SourceDataForFigure3C,3G,3H/3G/Blot_IP_anti-GFP_Marker.tif]

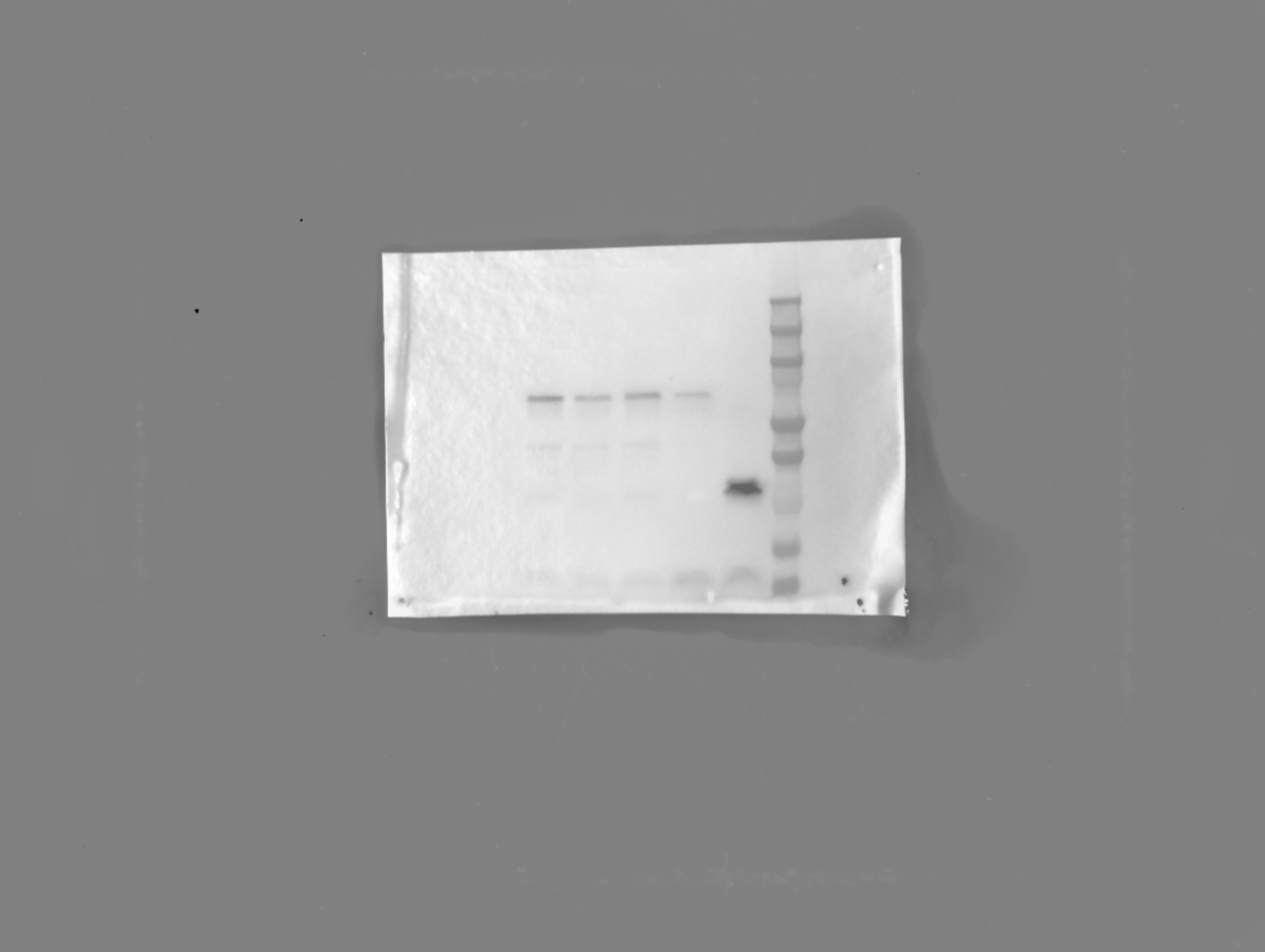

Supplement: Supplementary file 2 — Source data Fig. 3 [file 44319_2024_266_MOESM2_ESM.zip › EMBOR-2024-59287-SourceDataForFigure3C,3G,3H/3G/Blot_IP_anti-GFP-merged.tif]

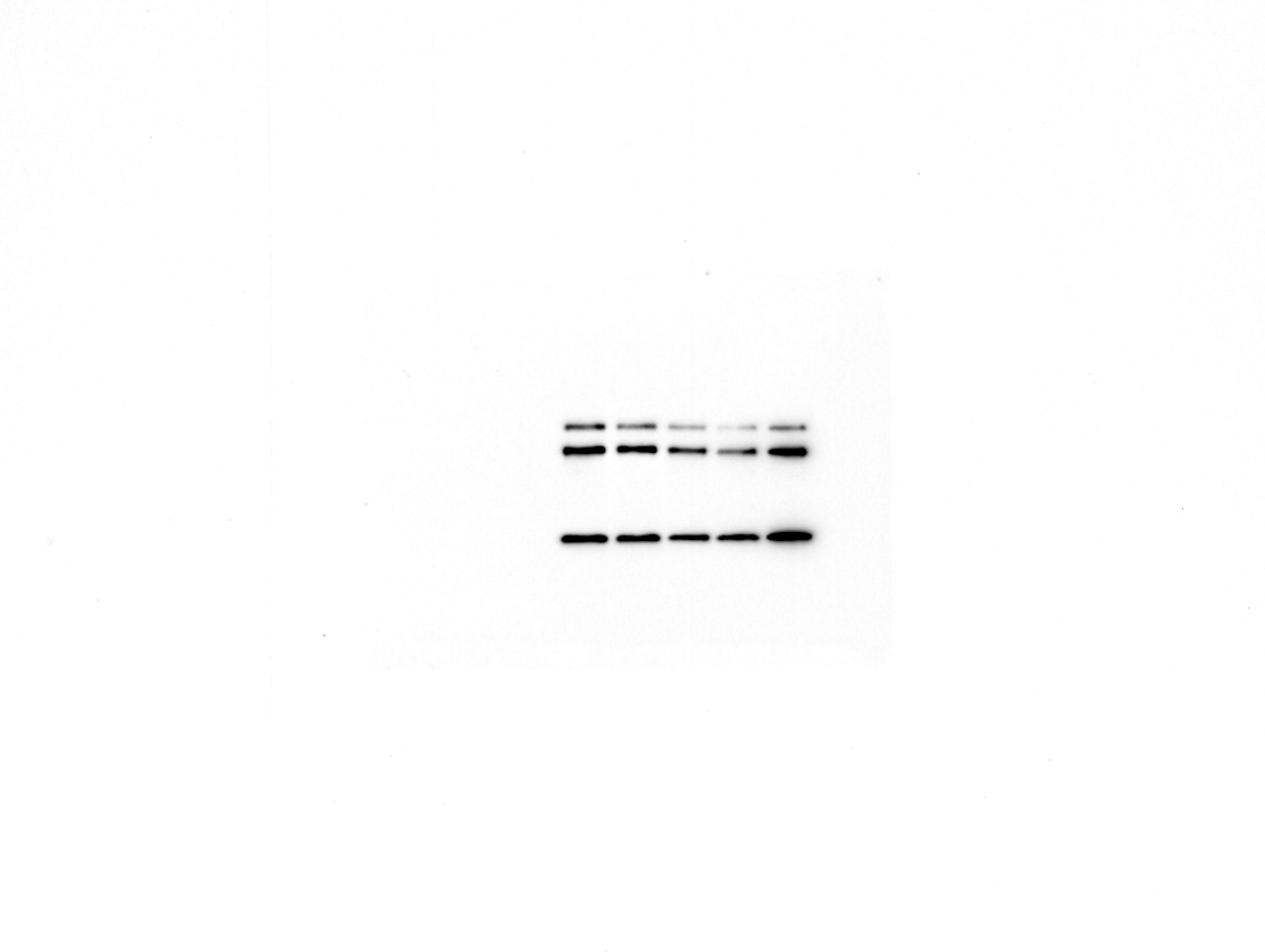

Supplement: Supplementary file 2 — Source data Fig. 3 [file 44319_2024_266_MOESM2_ESM.zip › EMBOR-2024-59287-SourceDataForFigure3C,3G,3H/3G/Blot_Input_anti-MOSPD2.tif]

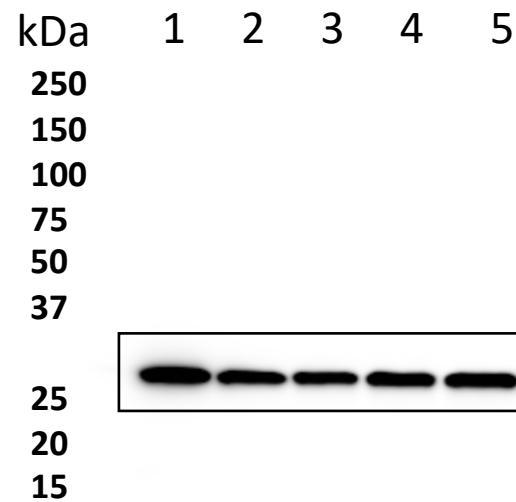

Anti-VAPB

- 1 GFP
- 2 *CbEPF1*-wt-GFP
- 3 *CbEPF1*-F1mt-GFP
- 4 *CbEPF1*-F2mt-GFP
- 5 *CbEPF1*-F3mt-GFP

Supplement: Supplementary file 2 — Source data Fig. 3 [file 44319_2024_266_MOESM2_ESM.zip › EMBOR-2024-59287-SourceDataForFigure3C,3G,3H/3G/Blot_Input_anti-VAPB-annotated.pdf]

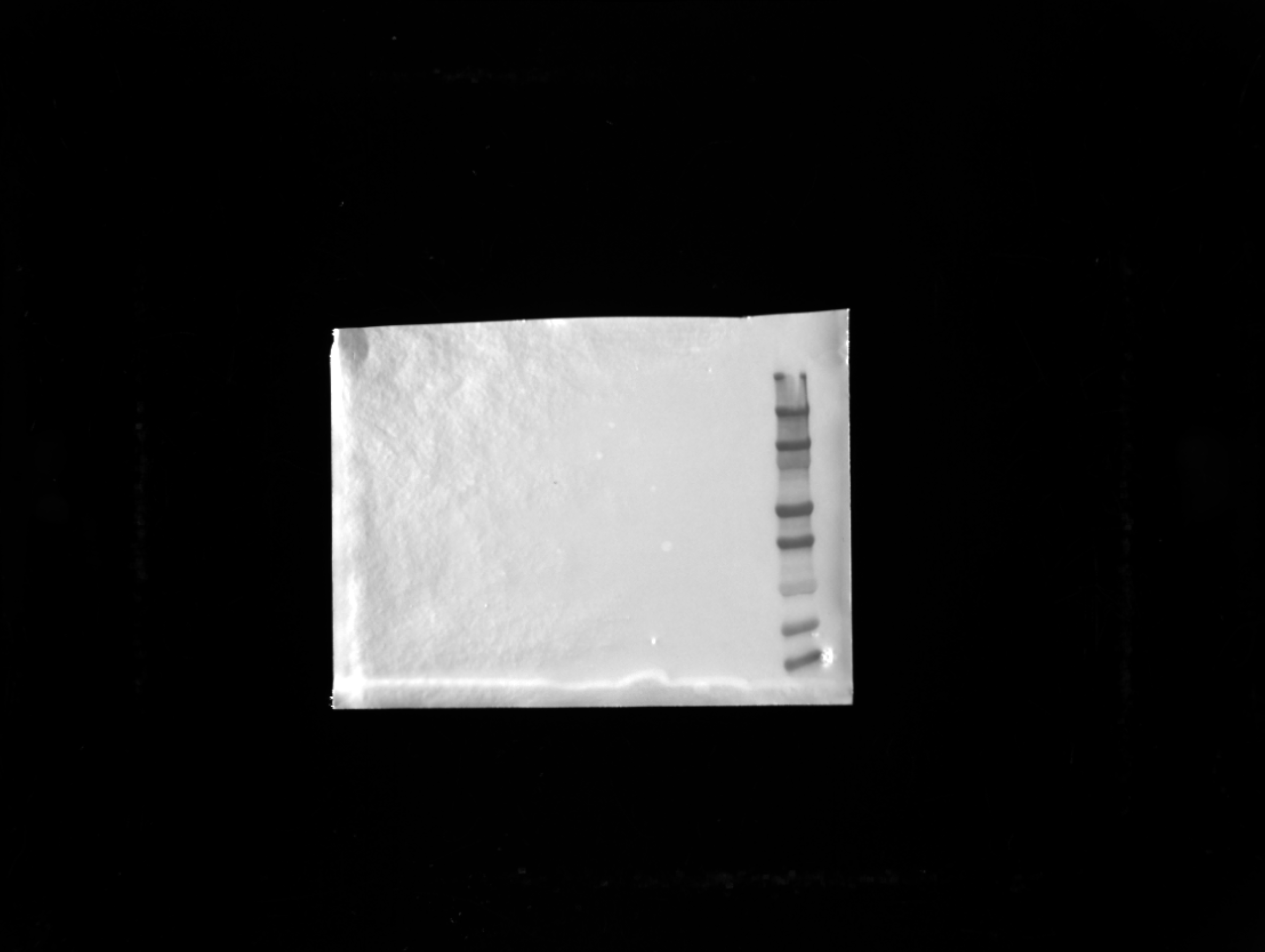

Supplement: Supplementary file 2 — Source data Fig. 3 [file 44319_2024_266_MOESM2_ESM.zip › EMBOR-2024-59287-SourceDataForFigure3C,3G,3H/3G/Blot_Input_anti-VAPB-Marker.tif]

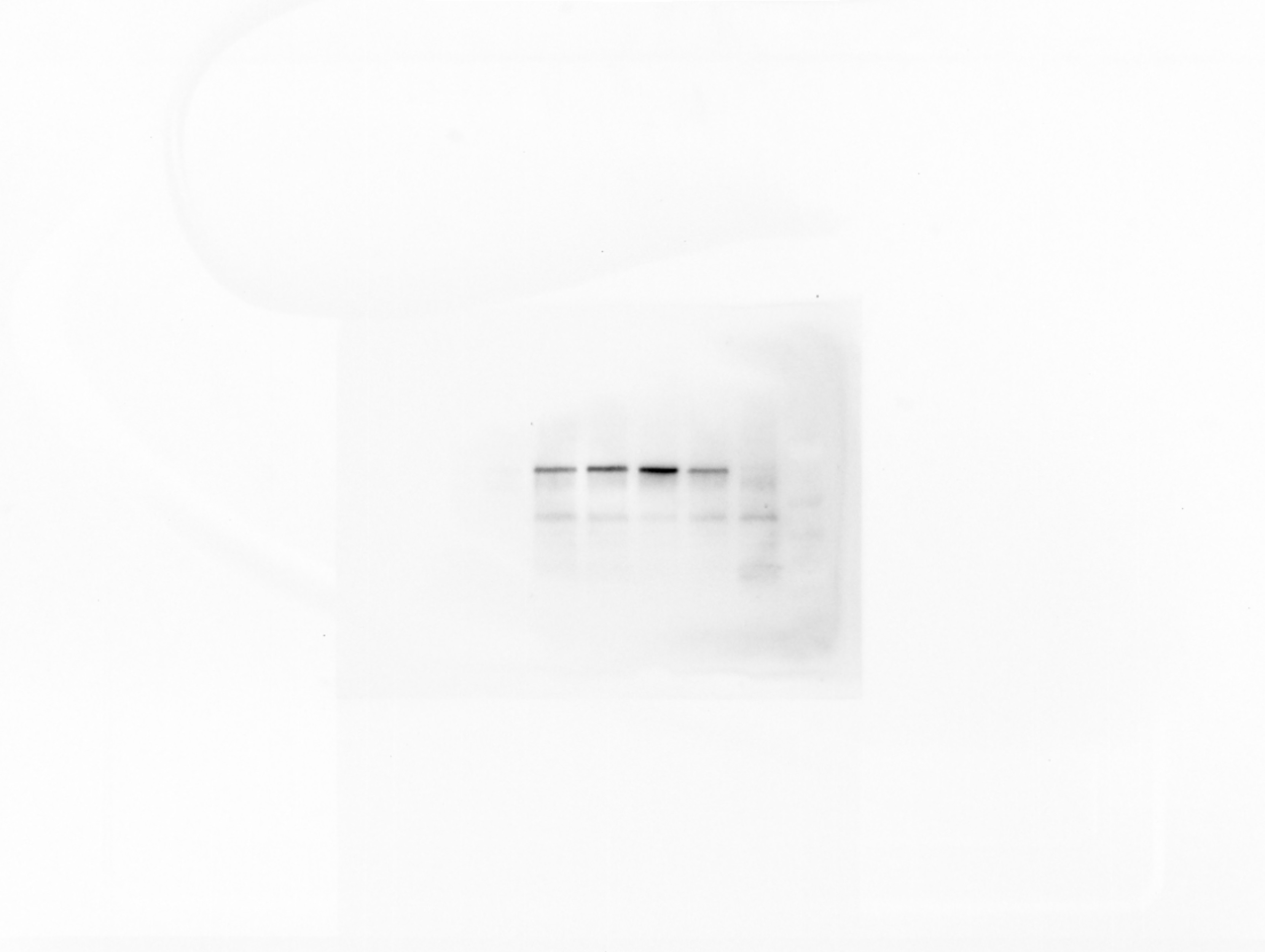

Supplement: Supplementary file 2 — Source data Fig. 3 [file 44319_2024_266_MOESM2_ESM.zip › EMBOR-2024-59287-SourceDataForFigure3C,3G,3H/3G/Blot_Input_anti-GFP.tif]

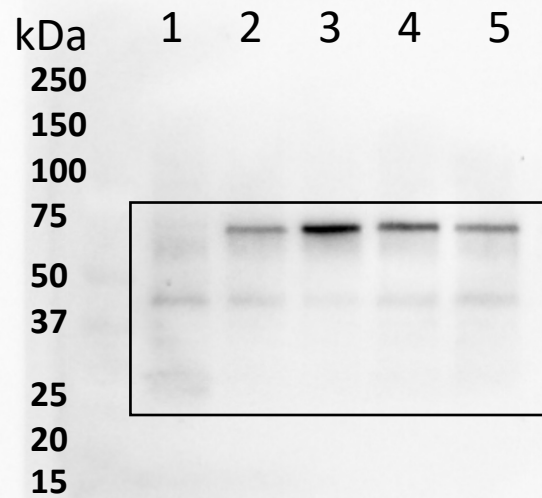

Anti-GFP

- 1 GFP
- 2 *CbEPF1*-wt-GFP
- 3 *CbEPF1*-F1mt-GFP
- 4 *CbEPF1*-F2mt-GFP
- 5 *CbEPF1*-F3mt-GFP

Supplement: Supplementary file 2 — Source data Fig. 3 [file 44319_2024_266_MOESM2_ESM.zip › EMBOR-2024-59287-SourceDataForFigure3C,3G,3H/3G/Blot_Input_anti-GFP-Annotated.pdf]

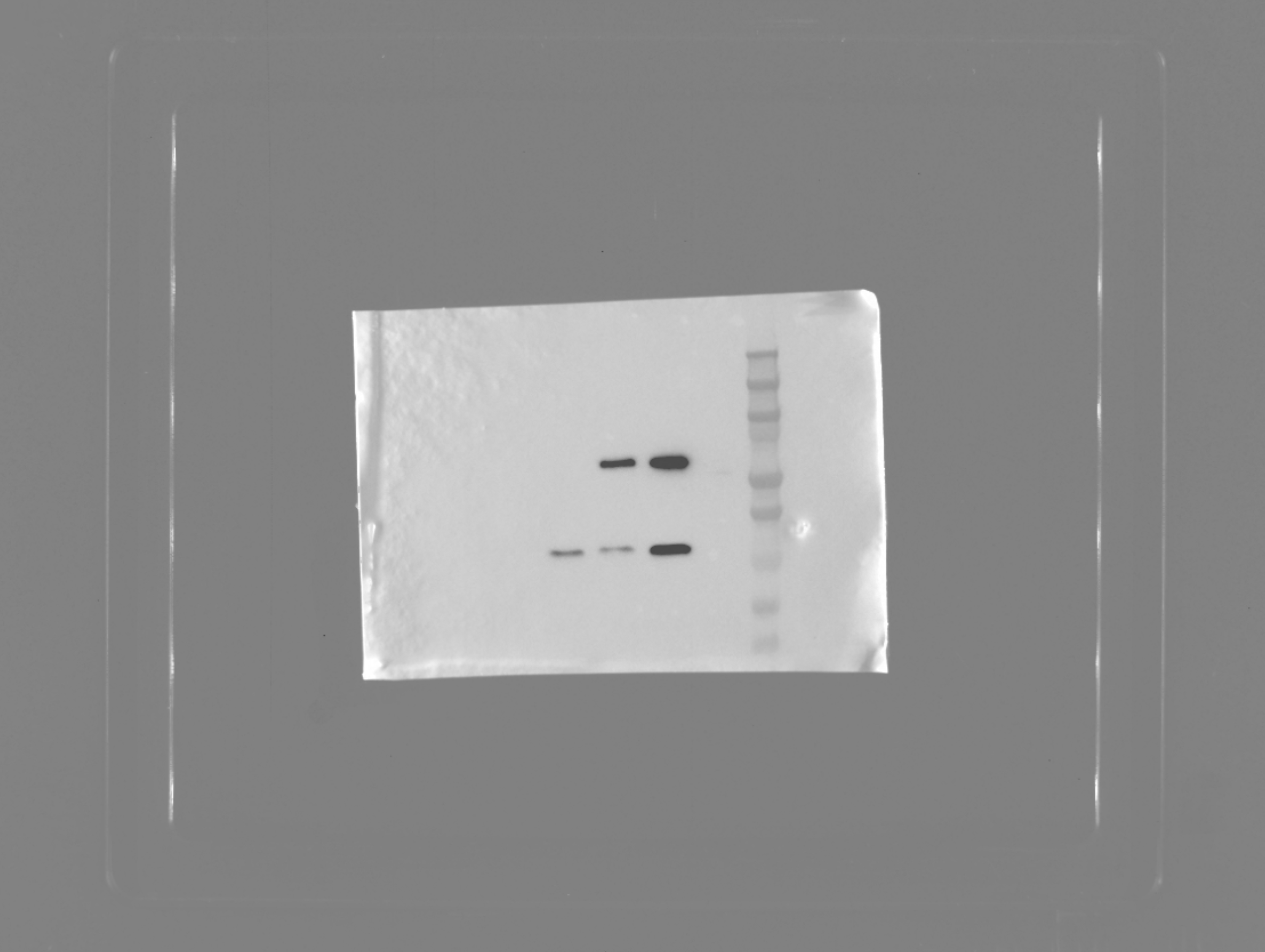

Supplement: Supplementary file 2 — Source data Fig. 3 [file 44319_2024_266_MOESM2_ESM.zip › EMBOR-2024-59287-SourceDataForFigure3C,3G,3H/3G/Blot_IP_anti-MOSPD2_Merged.tif]

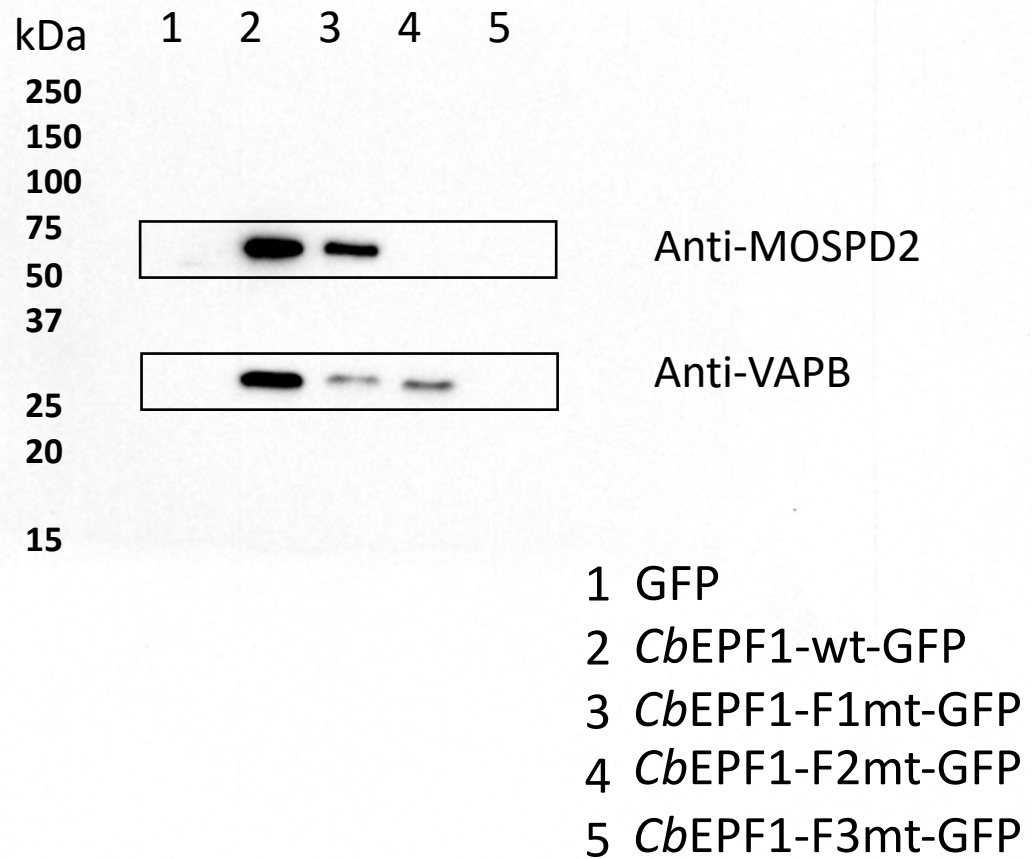

Supplement: Supplementary file 2 — Source data Fig. 3 [file 44319_2024_266_MOESM2_ESM.zip › EMBOR-2024-59287-SourceDataForFigure3C,3G,3H/3G/Blot_IP_anti-MOSPD2_Annotated.pdf]

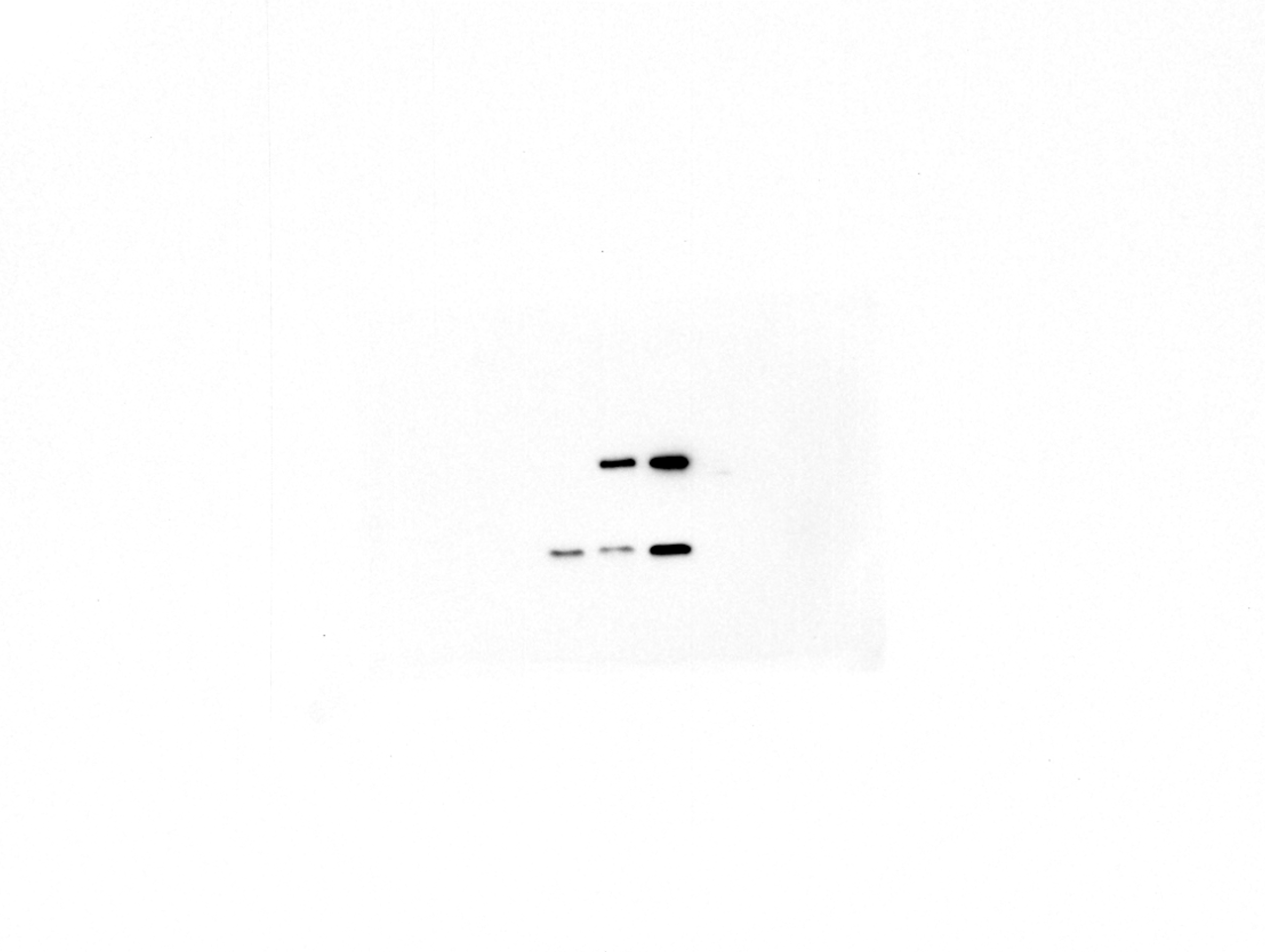

Supplement: Supplementary file 2 — Source data Fig. 3 [file 44319_2024_266_MOESM2_ESM.zip › EMBOR-2024-59287-SourceDataForFigure3C,3G,3H/3G/Blot_IP_anti-MOSPD2.tif]

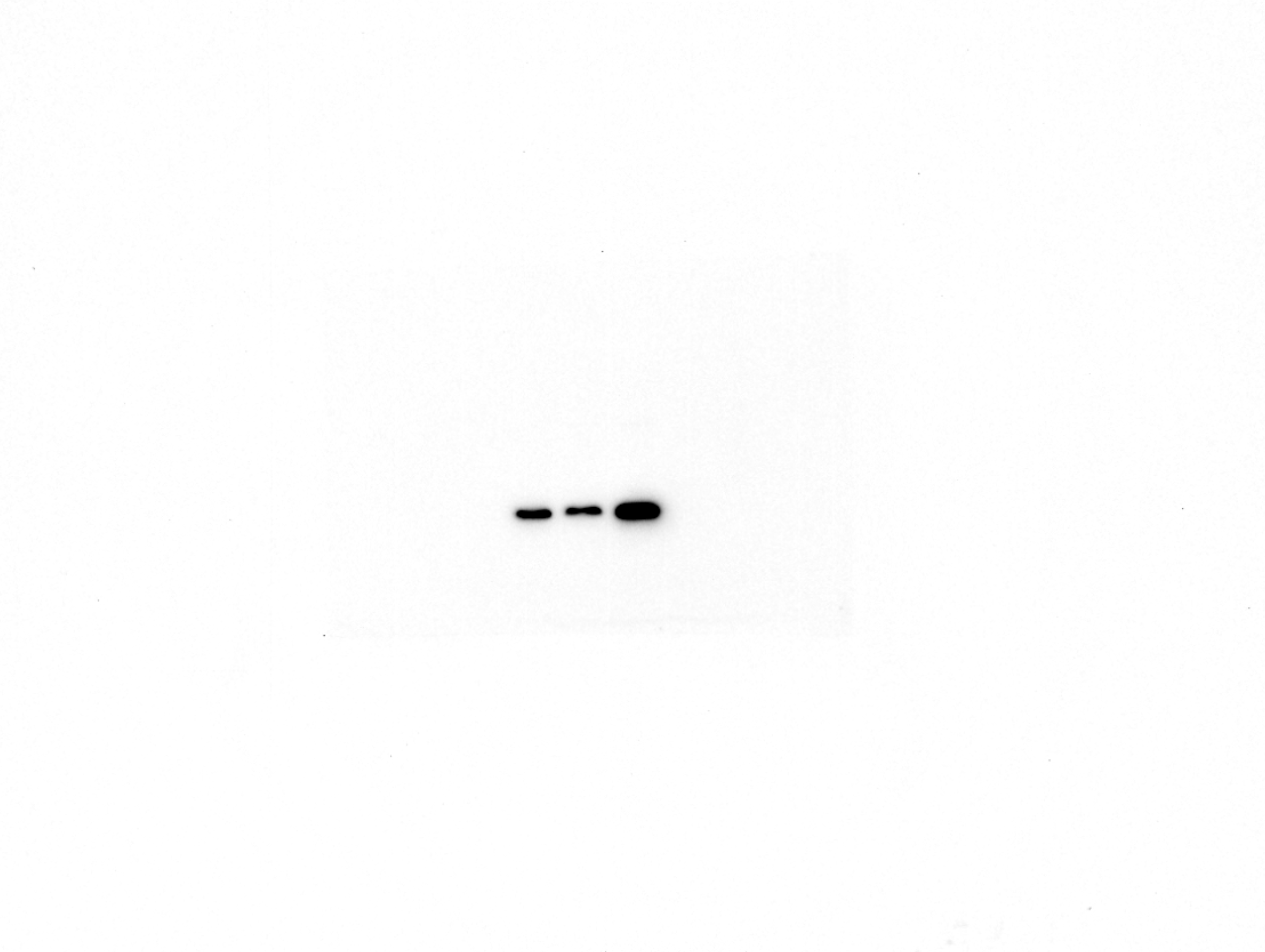

Supplement: Supplementary file 2 — Source data Fig. 3 [file 44319_2024_266_MOESM2_ESM.zip › EMBOR-2024-59287-SourceDataForFigure3C,3G,3H/3G/Blot_IP_anti-VAPB.tif]

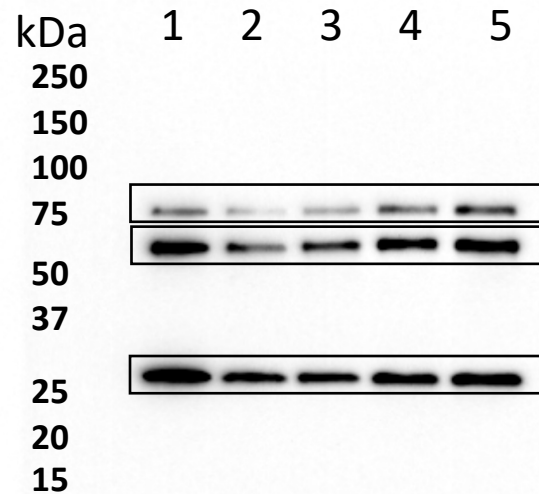

Non-specific  
Anti-MOSPD2

Anti-VAPB

- 1 GFP
- 2 *CbEPF1*-wt-GFP
- 3 *CbEPF1*-F1mt-GFP
- 4 *CbEPF1*-F2mt-GFP
- 5 *CbEPF1*-F3mt-GFP

Supplement: Supplementary file 2 — Source data Fig. 3 [file 44319_2024_266_MOESM2_ESM.zip › EMBOR-2024-59287-SourceDataForFigure3C,3G,3H/3G/Blot_Input_anti-MOSPD2-Annotated.pdf]

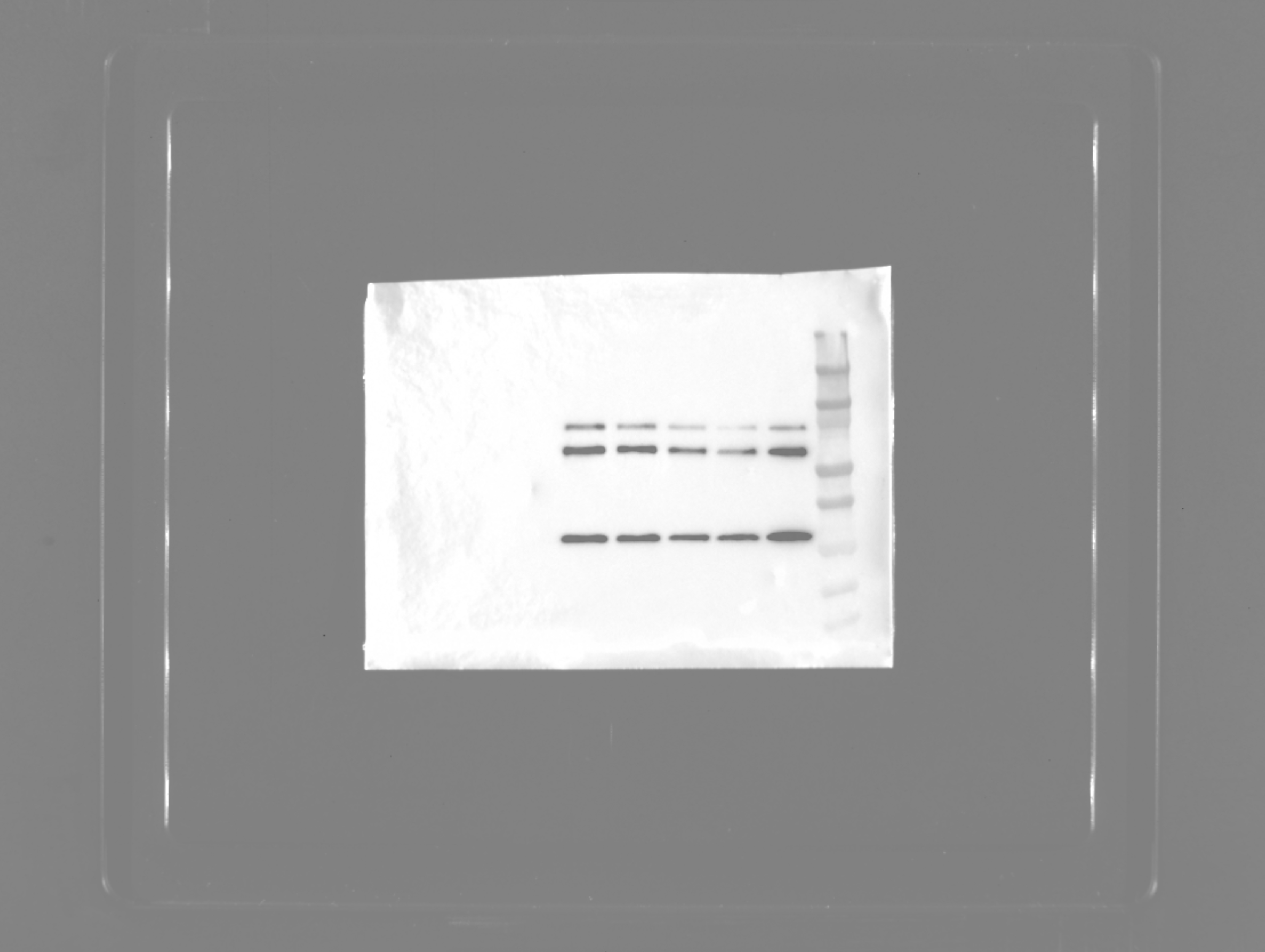

Supplement: Supplementary file 2 — Source data Fig. 3 [file 44319_2024_266_MOESM2_ESM.zip › EMBOR-2024-59287-SourceDataForFigure3C,3G,3H/3G/Blot_Input_anti-MOSPD2-Merged.tif]

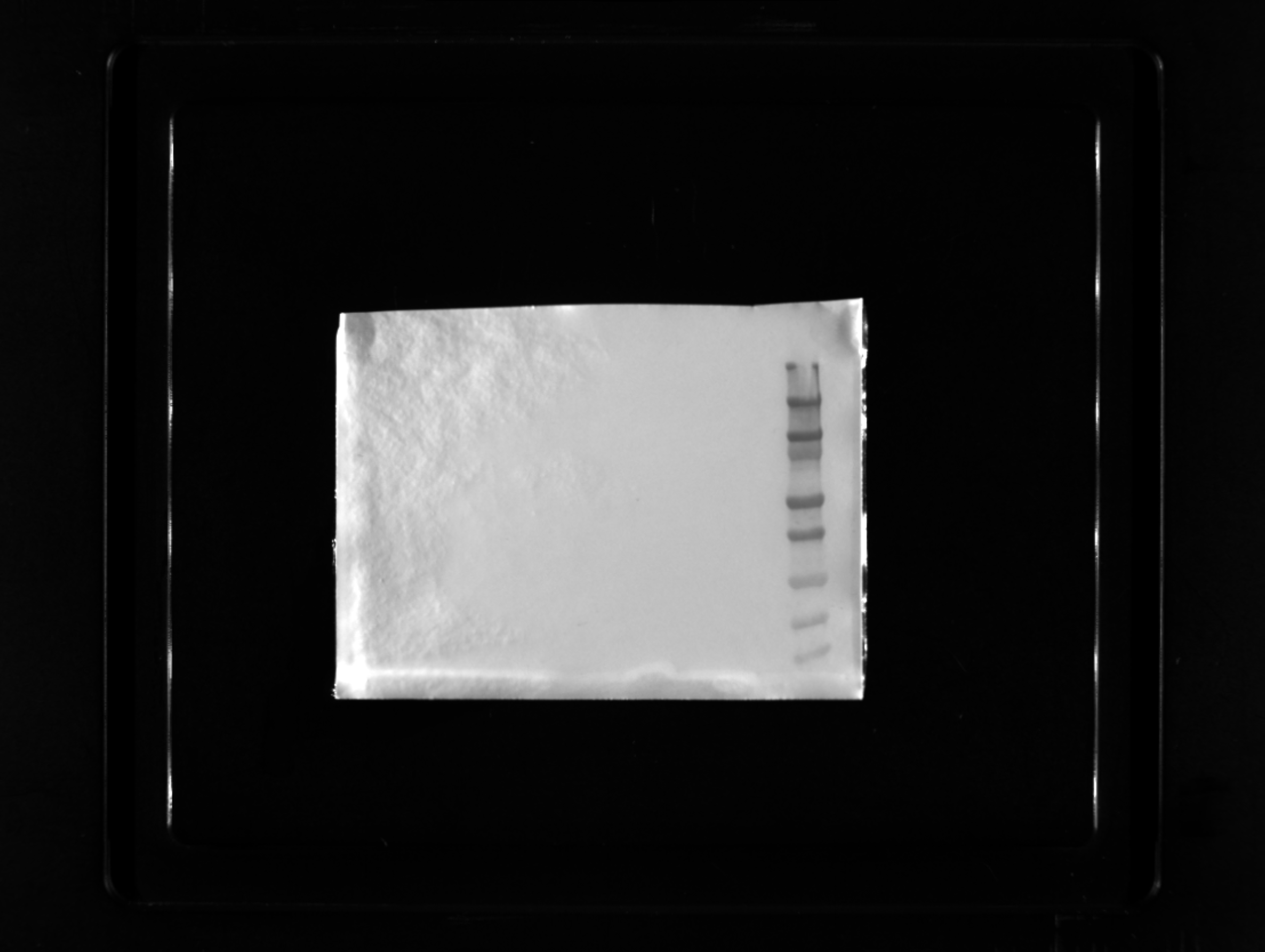

Supplement: Supplementary file 2 — Source data Fig. 3 [file 44319_2024_266_MOESM2_ESM.zip › EMBOR-2024-59287-SourceDataForFigure3C,3G,3H/3G/Blot_Input_anti-GFP-Marker.tif]

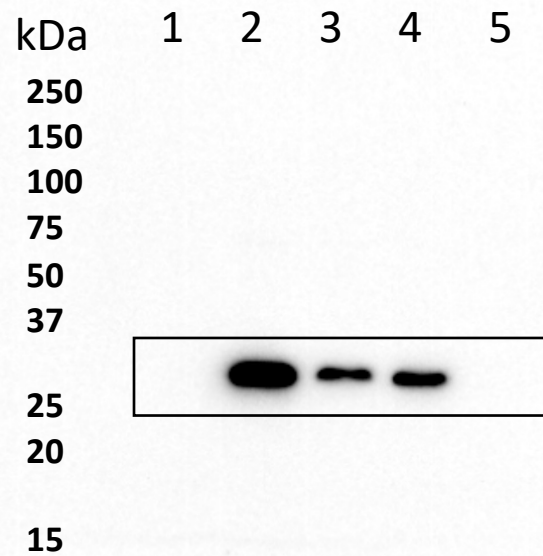

Anti-VAPB

- 1 GFP
- 2 *CbEPF1*-wt-GFP
- 3 *CbEPF1*-F1mt-GFP
- 4 *CbEPF1*-F2mt-GFP
- 5 *CbEPF1*-F3mt-GFP

Supplement: Supplementary file 2 — Source data Fig. 3 [file 44319_2024_266_MOESM2_ESM.zip › EMBOR-2024-59287-SourceDataForFigure3C,3G,3H/3G/Blot_IP_anti-VAPB_annotated.pdf]

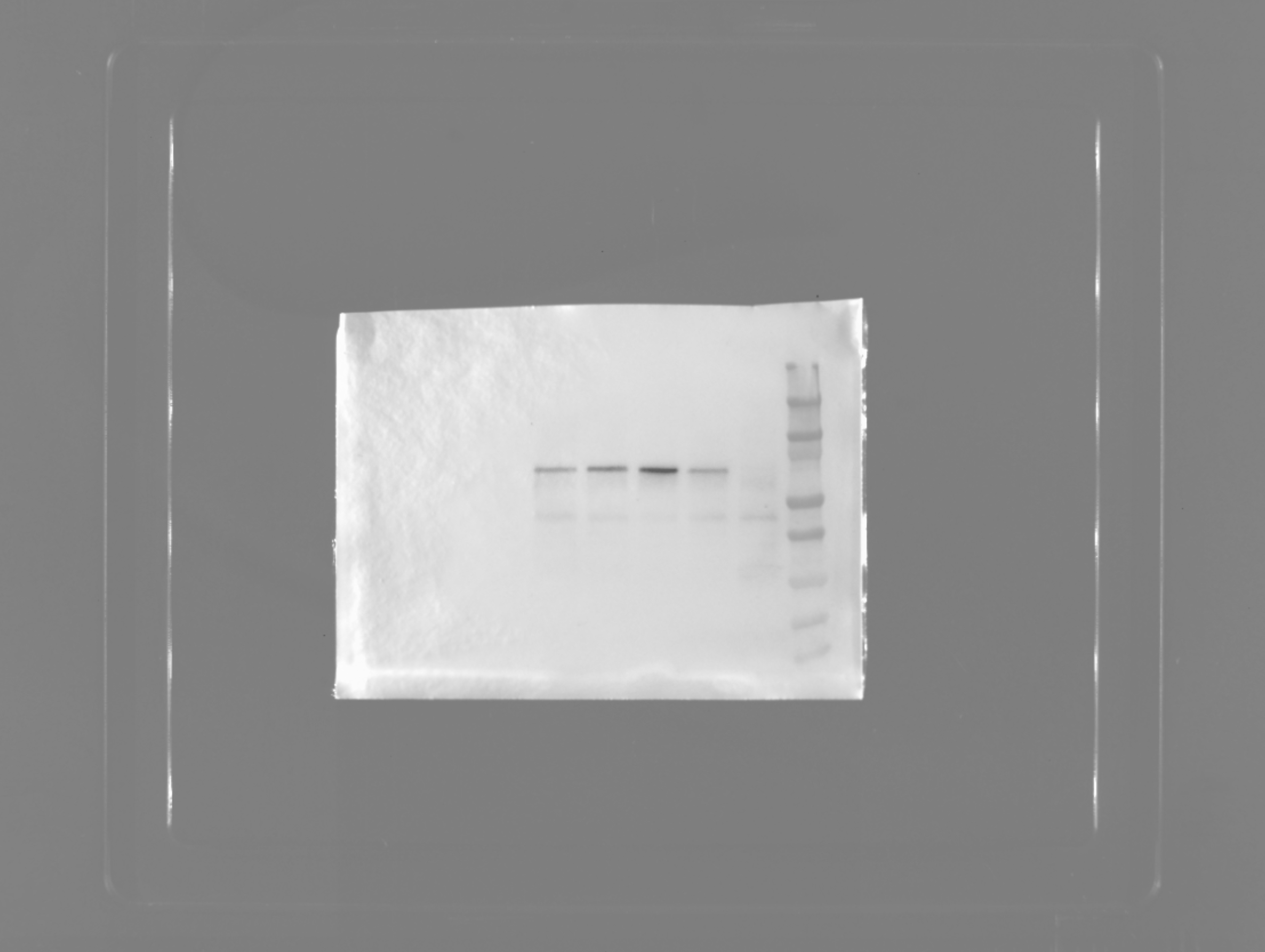

Supplement: Supplementary file 2 — Source data Fig. 3 [file 44319_2024_266_MOESM2_ESM.zip › EMBOR-2024-59287-SourceDataForFigure3C,3G,3H/3G/Blot_Input_anti-GFP-Merged.tif]

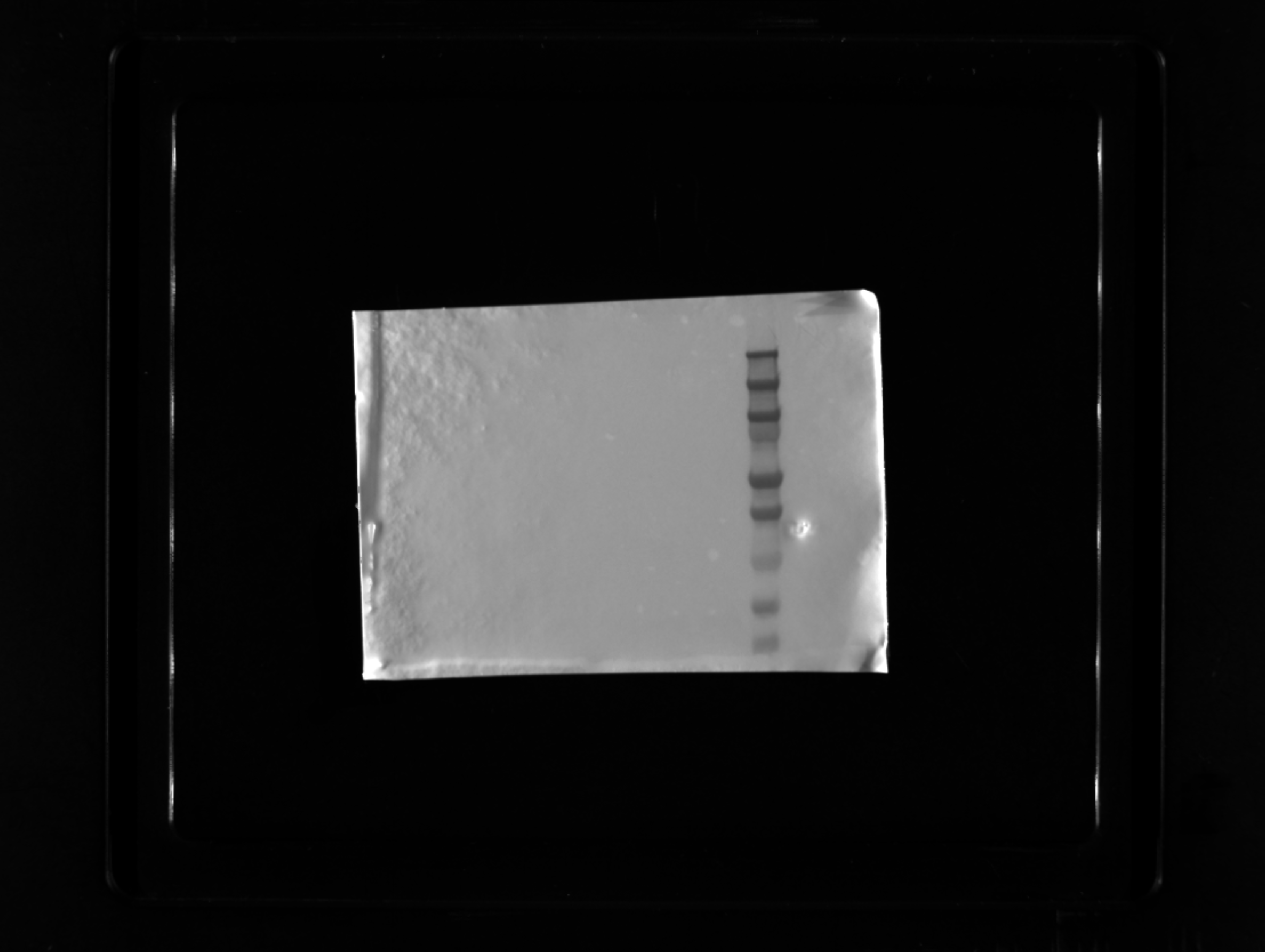

Supplement: Supplementary file 2 — Source data Fig. 3 [file 44319_2024_266_MOESM2_ESM.zip › EMBOR-2024-59287-SourceDataForFigure3C,3G,3H/3G/Blot_IP_anti-MOSPD2-Marker.tif]

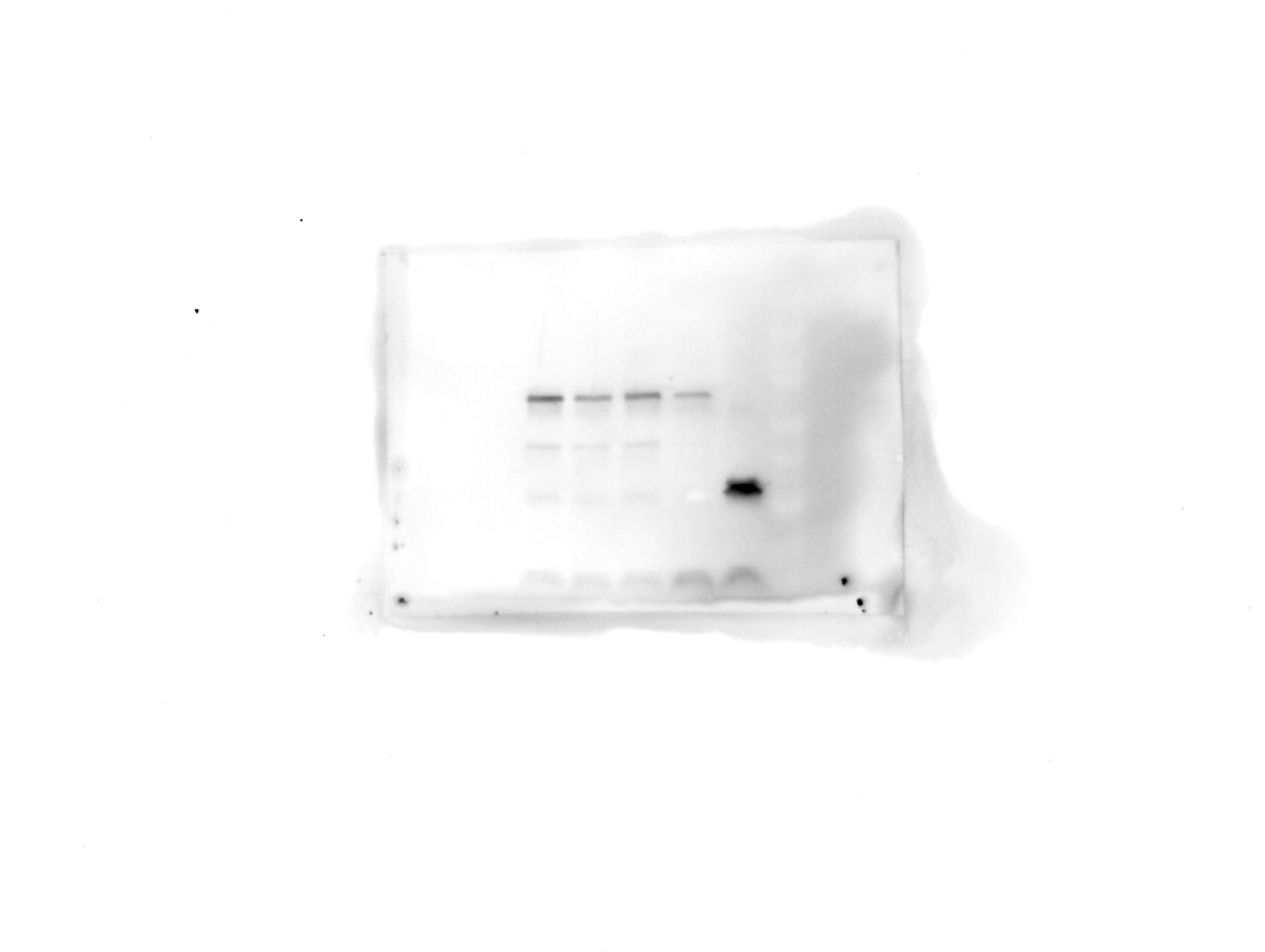

Supplement: Supplementary file 2 — Source data Fig. 3 [file 44319_2024_266_MOESM2_ESM.zip › EMBOR-2024-59287-SourceDataForFigure3C,3G,3H/3G/Blot_IP_anti-GFP.tif]

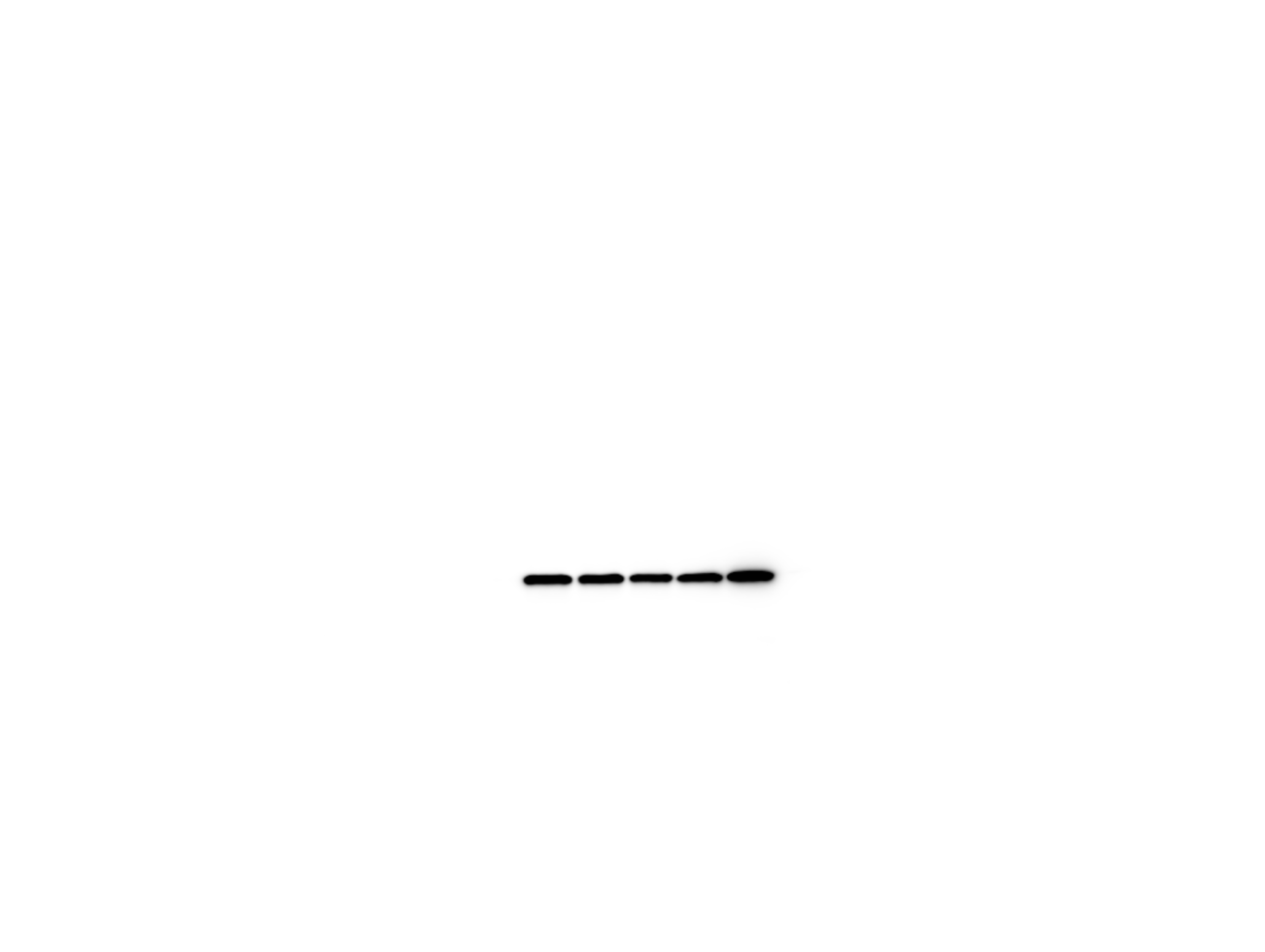

Supplement: Supplementary file 2 — Source data Fig. 3 [file 44319_2024_266_MOESM2_ESM.zip › EMBOR-2024-59287-SourceDataForFigure3C,3G,3H/3G/Blot_Input_anti-VAPB-.tif]

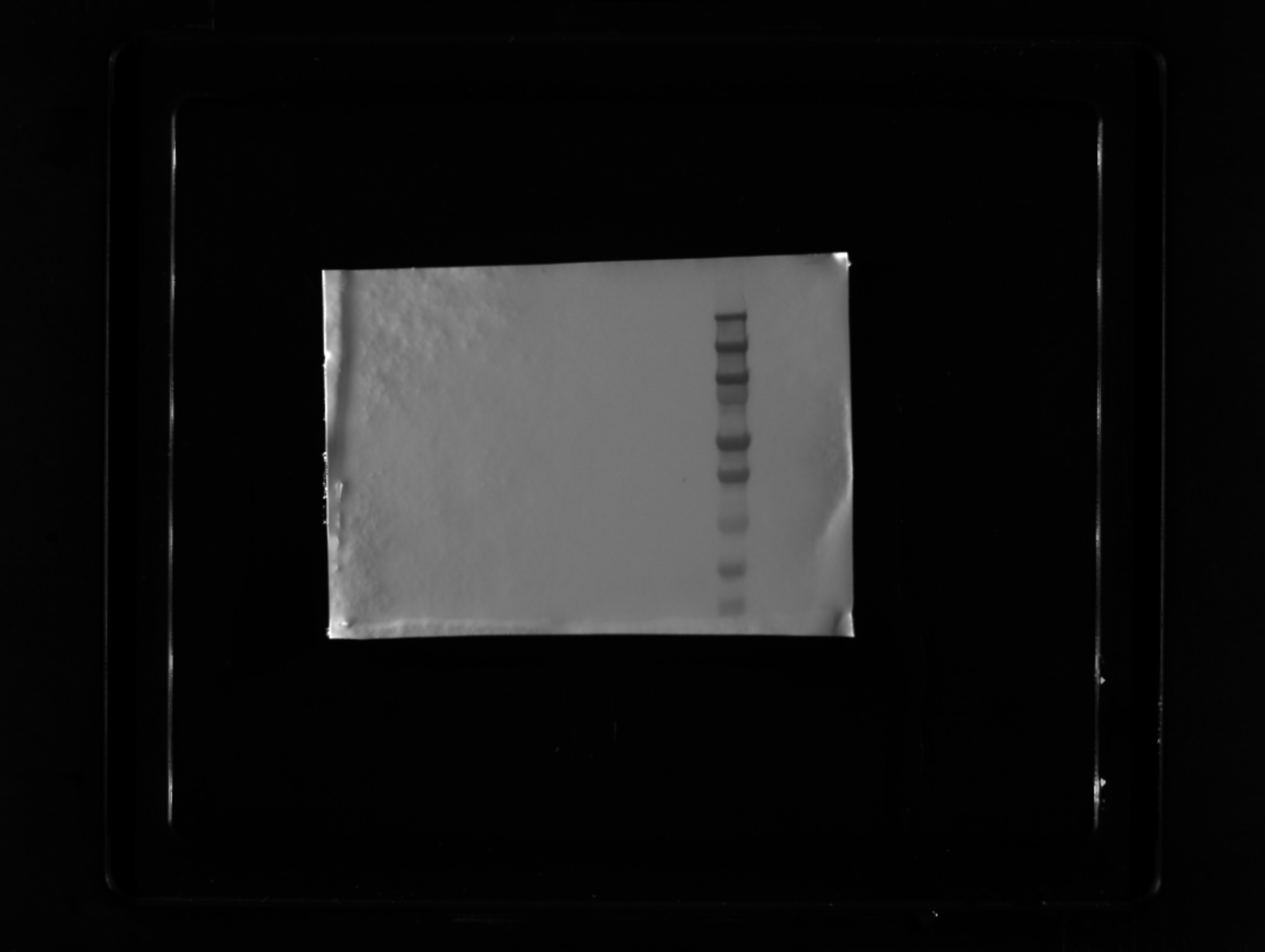

Supplement: Supplementary file 2 — Source data Fig. 3 [file 44319_2024_266_MOESM2_ESM.zip › EMBOR-2024-59287-SourceDataForFigure3C,3G,3H/3G/Blot_IP_anti-VAPB-marker.tif]

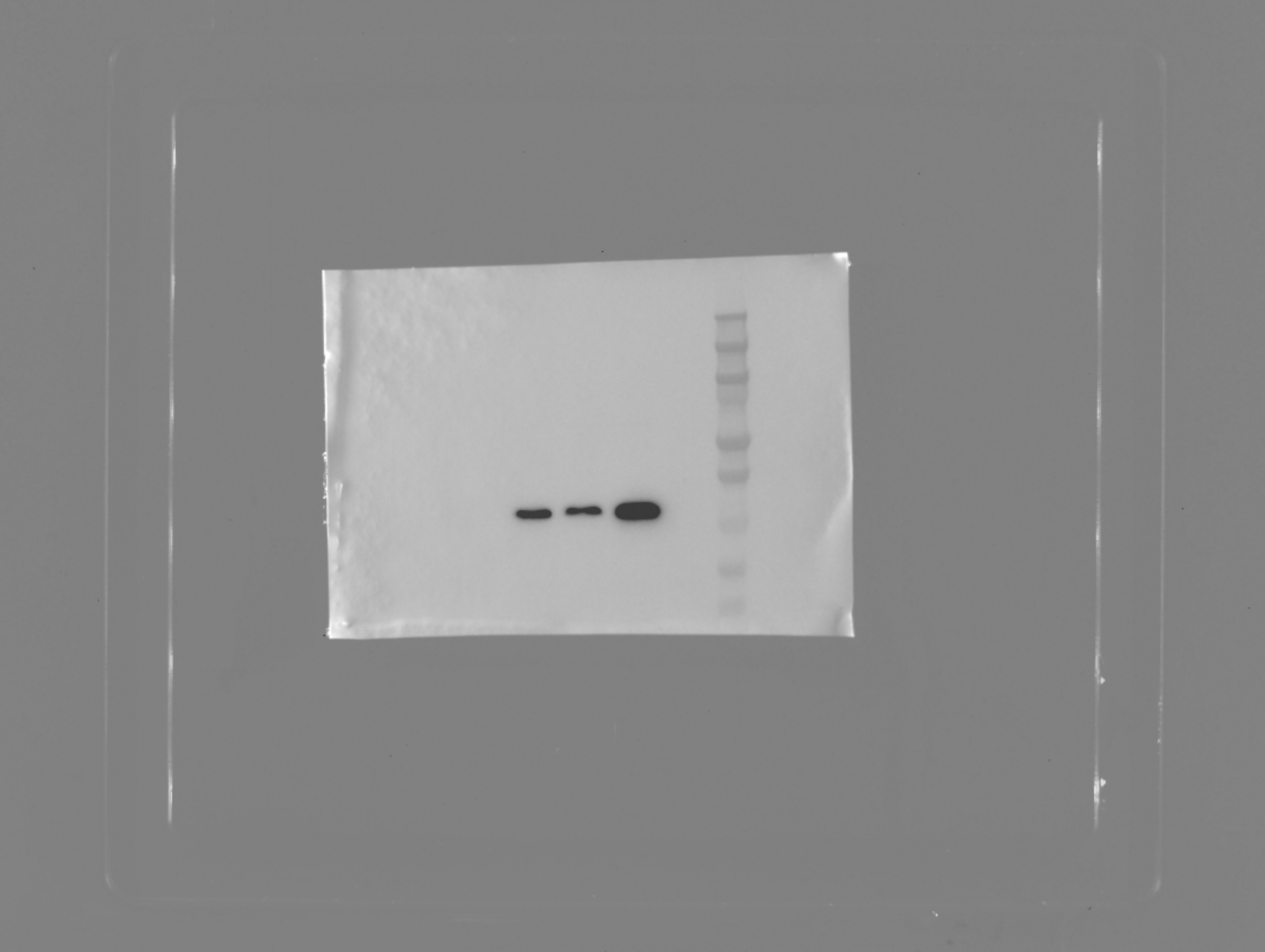

Supplement: Supplementary file 2 — Source data Fig. 3 [file 44319_2024_266_MOESM2_ESM.zip › EMBOR-2024-59287-SourceDataForFigure3C,3G,3H/3G/Blot_IP_anti-VAPB-Merged.tif]

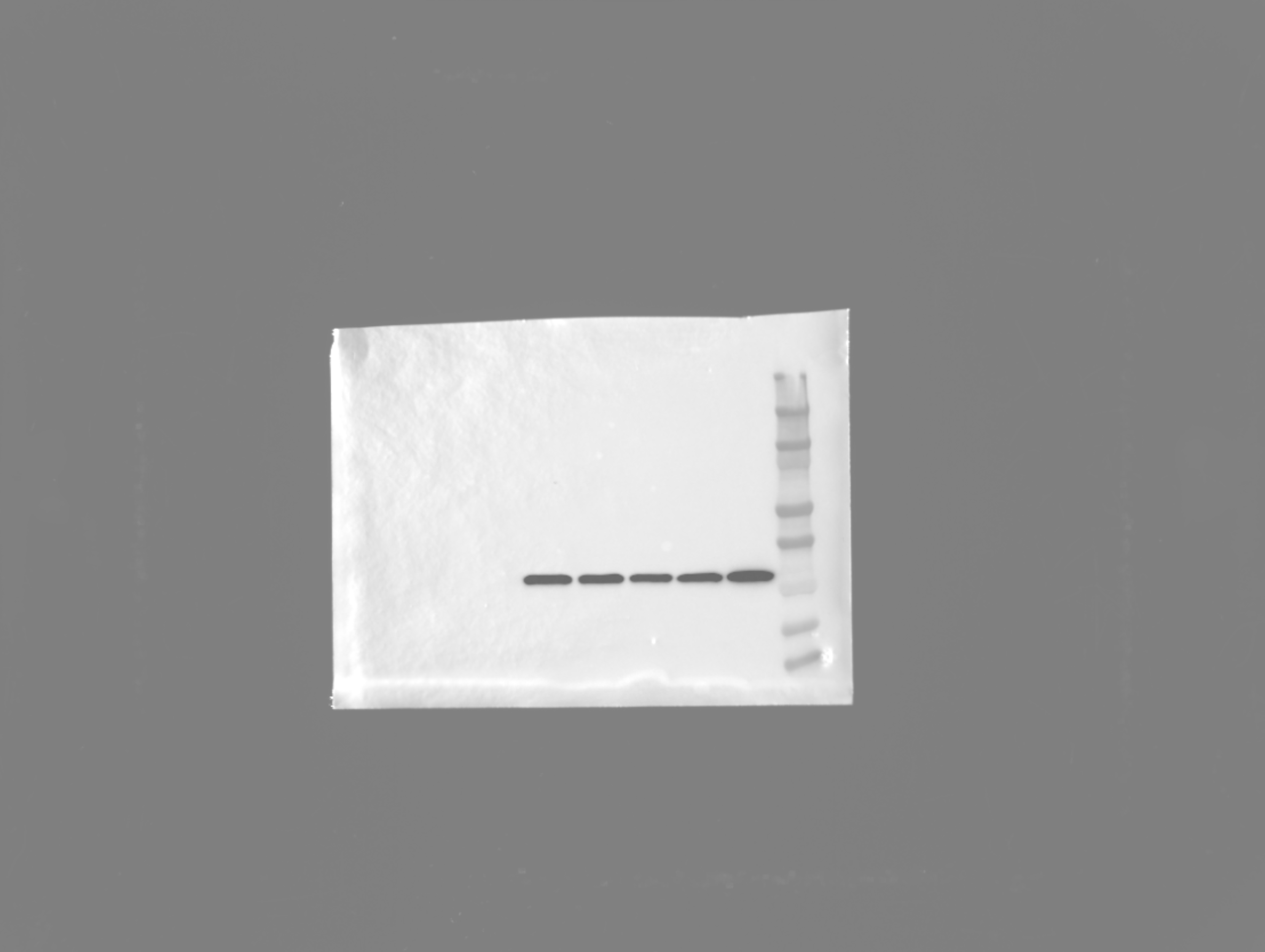

Supplement: Supplementary file 2 — Source data Fig. 3 [file 44319_2024_266_MOESM2_ESM.zip › EMBOR-2024-59287-SourceDataForFigure3C,3G,3H/3G/Blot_Input_anti-VAPB-Merged.tif]

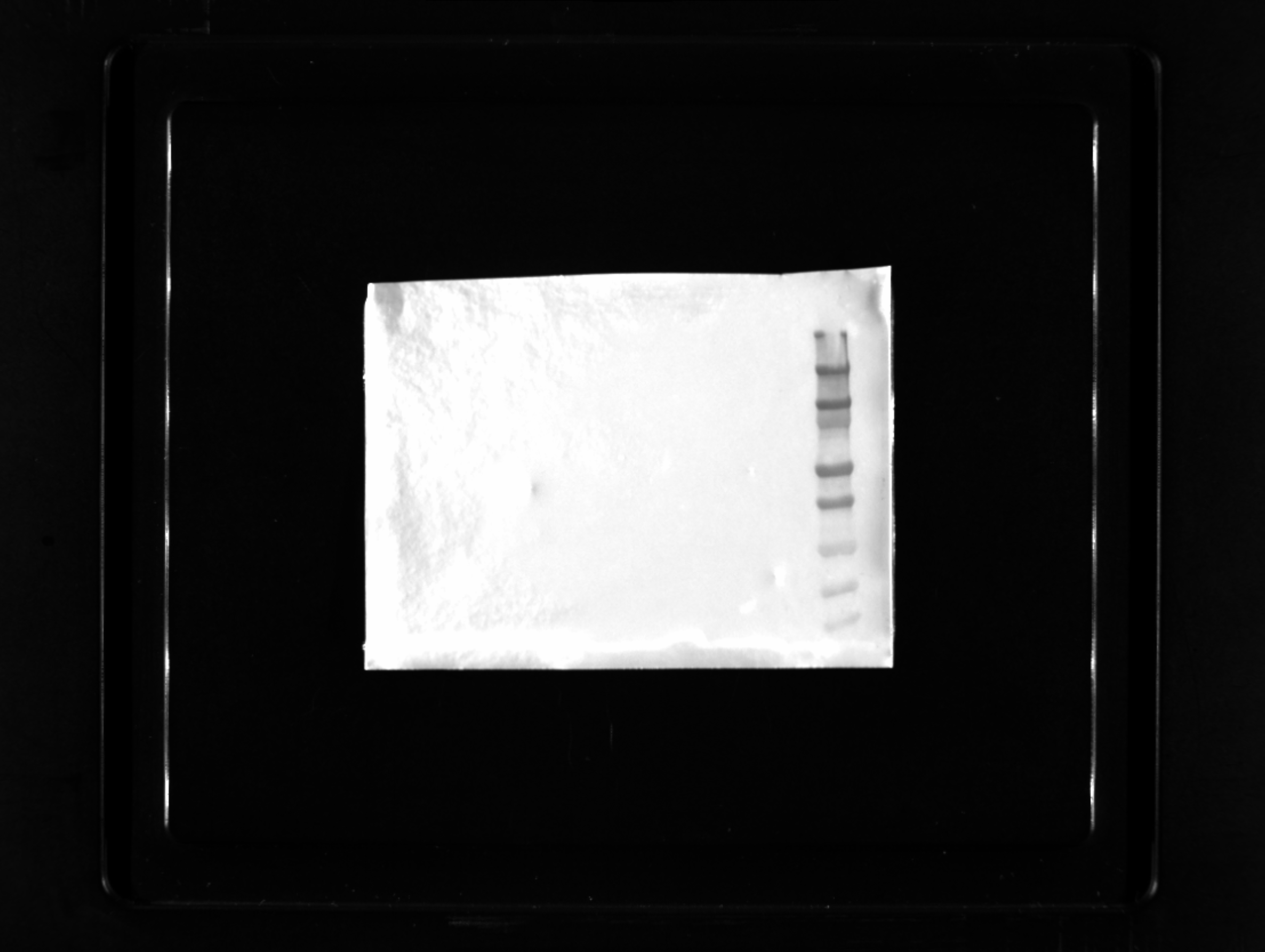

Supplement: Supplementary file 2 — Source data Fig. 3 [file 44319_2024_266_MOESM2_ESM.zip › EMBOR-2024-59287-SourceDataForFigure3C,3G,3H/3G/Blot_Input_anti-MOSPD2-Marker.tif]

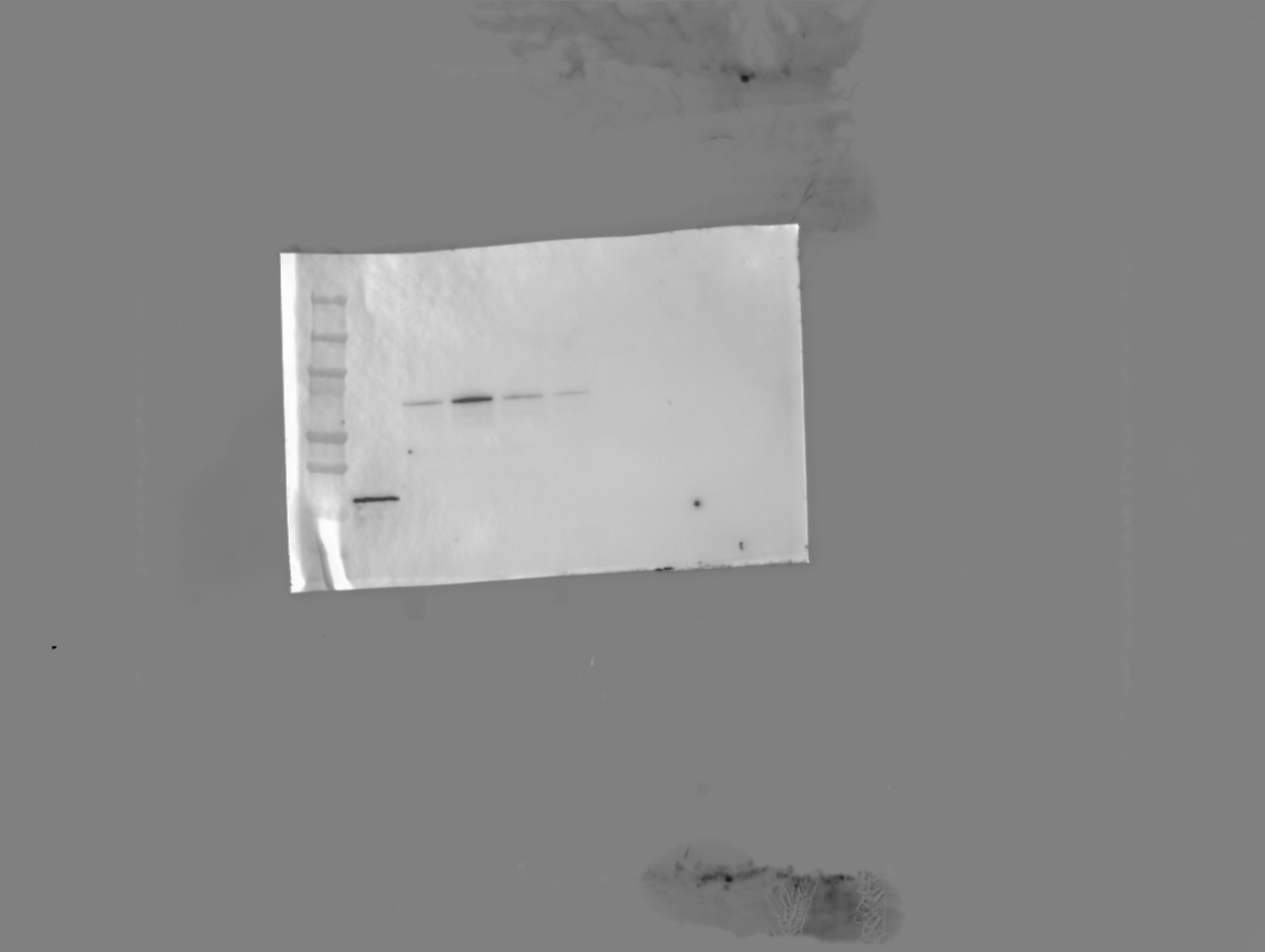

Supplement: Supplementary file 2 — Source data Fig. 3 [file 44319_2024_266_MOESM2_ESM.zip › EMBOR-2024-59287-SourceDataForFigure3C,3G,3H/3H/Blot_Input_anti-GFP-Merged.tif]

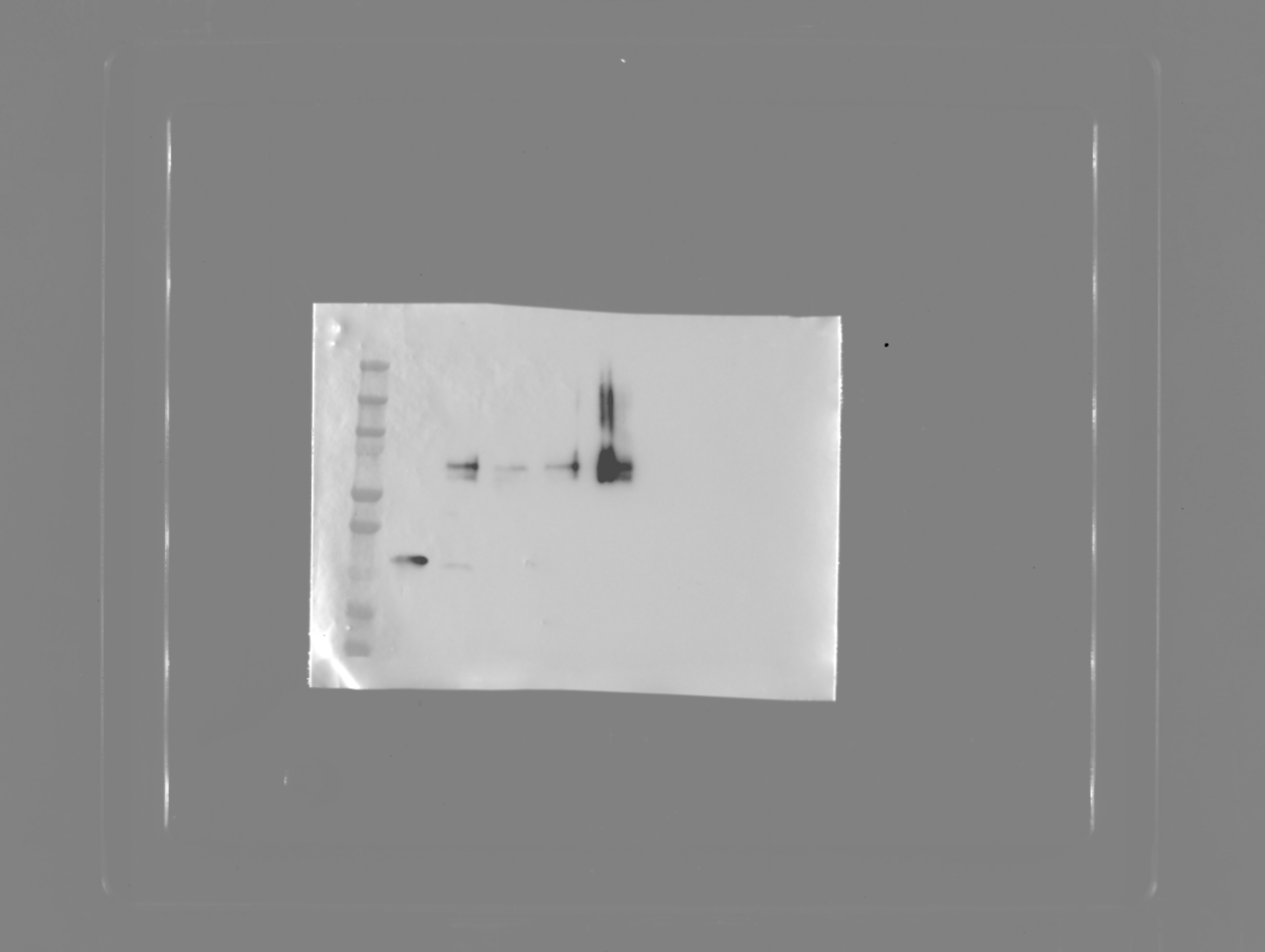

Supplement: Supplementary file 2 — Source data Fig. 3 [file 44319_2024_266_MOESM2_ESM.zip › EMBOR-2024-59287-SourceDataForFigure3C,3G,3H/3H/Blot_IP_Anti-GFP-Merged.tif]

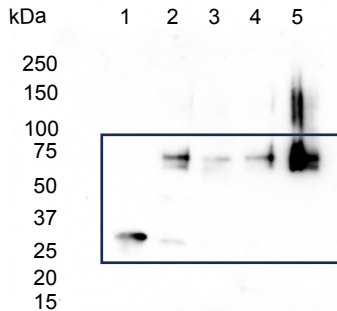

- 1 GFP
- 2 *CbEPF1*-wt-GFP
- 3 *CbEPF1*-F1mt-GFP
- 4 *CbEPF1*-F2mt-GFP
- 5 *CbEPF1*-F3mt-GFP

Supplement: Supplementary file 2 — Source data Fig. 3 [file 44319_2024_266_MOESM2_ESM.zip › EMBOR-2024-59287-SourceDataForFigure3C,3G,3H/3H/Blot_IP_Anti-GFP-Annotated.pdf]

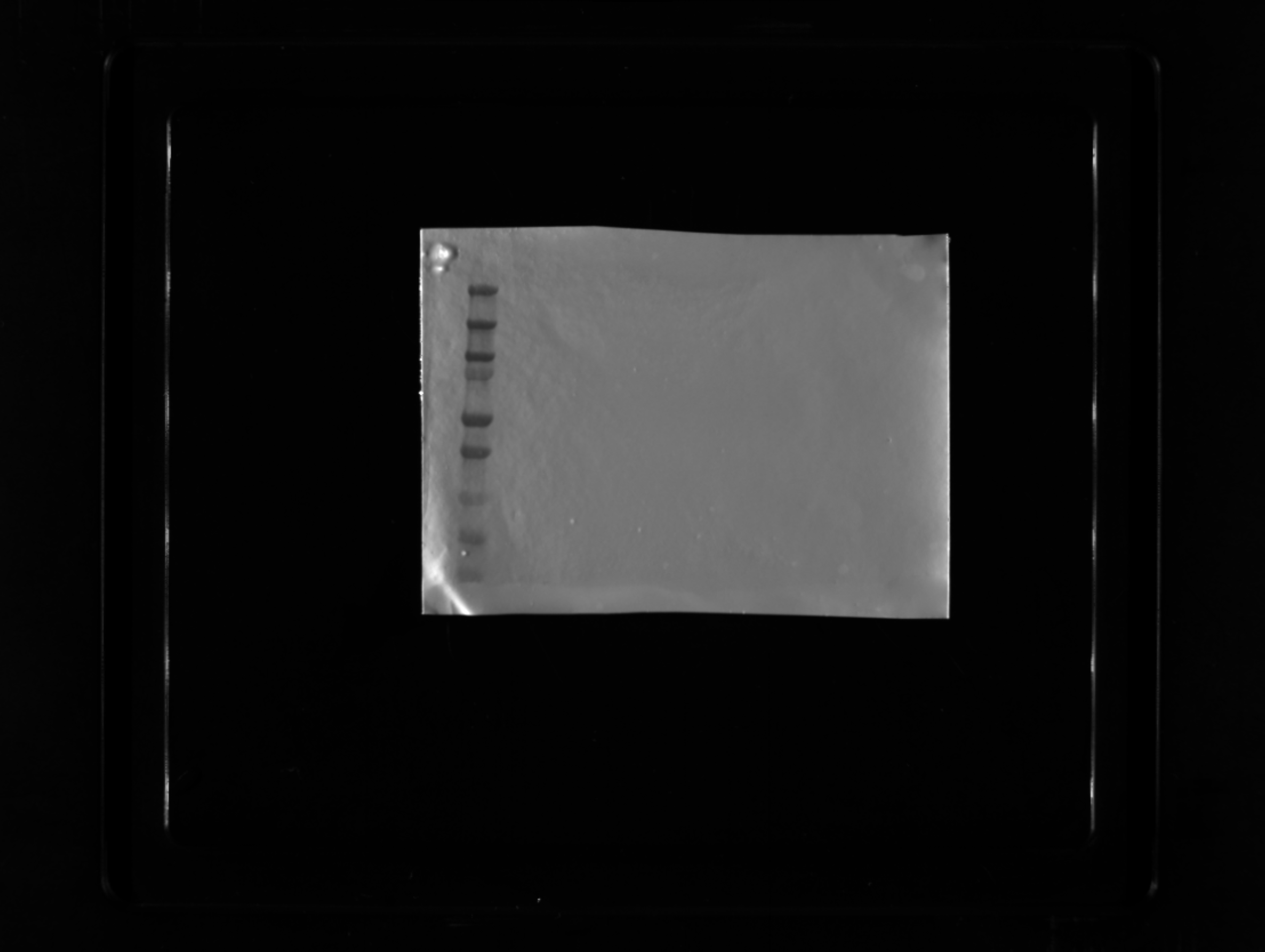

Supplement: Supplementary file 2 — Source data Fig. 3 [file 44319_2024_266_MOESM2_ESM.zip › EMBOR-2024-59287-SourceDataForFigure3C,3G,3H/3H/Blot_IP_anti-VAPA-Marker.tif]

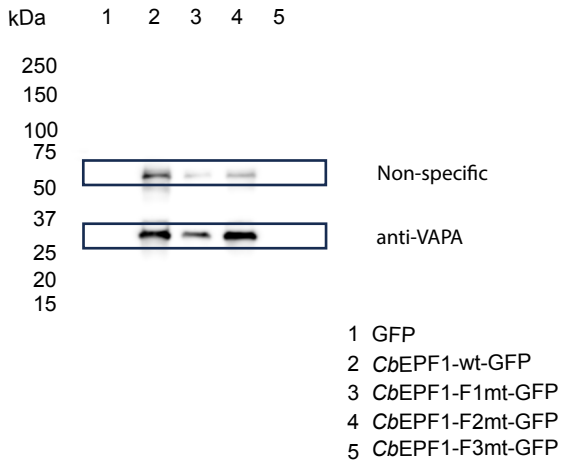

Supplement: Supplementary file 2 — Source data Fig. 3 [file 44319_2024_266_MOESM2_ESM.zip › EMBOR-2024-59287-SourceDataForFigure3C,3G,3H/3H/Blot_IP_anti-VAPA-Annotated.pdf]

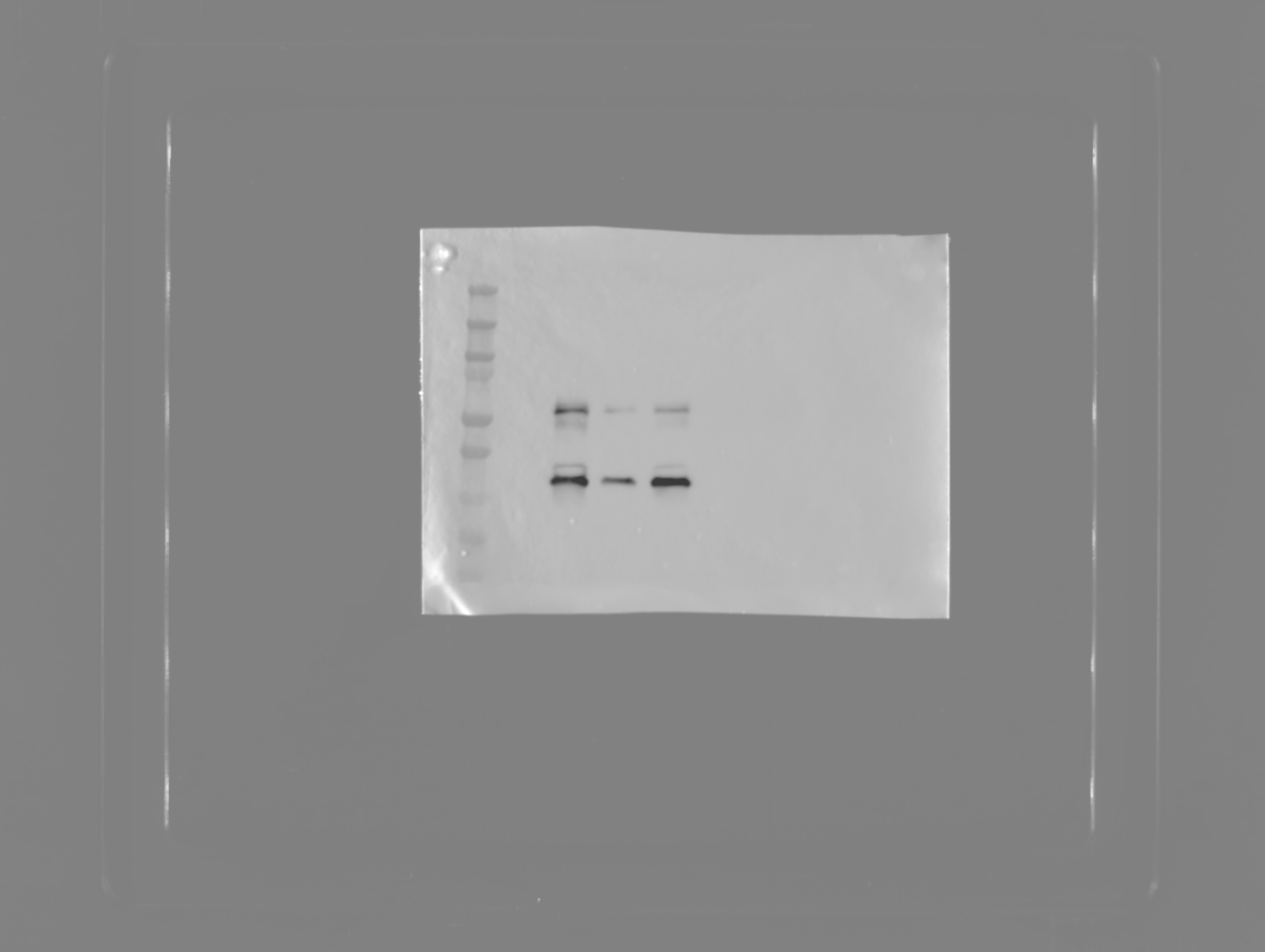

Supplement: Supplementary file 2 — Source data Fig. 3 [file 44319_2024_266_MOESM2_ESM.zip › EMBOR-2024-59287-SourceDataForFigure3C,3G,3H/3H/Blot_IP_anti-VAPA-Merged.tif]

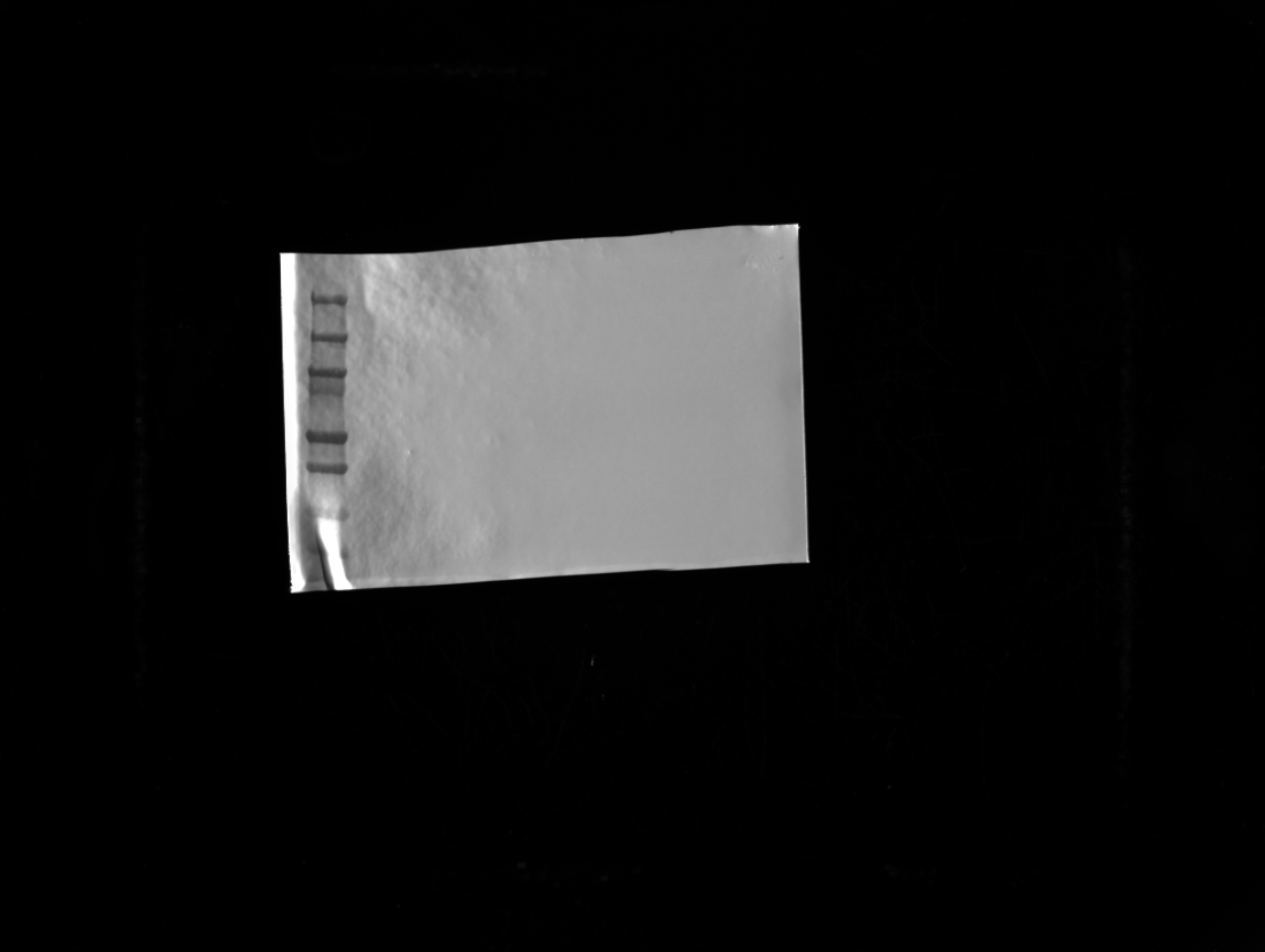

Supplement: Supplementary file 2 — Source data Fig. 3 [file 44319_2024_266_MOESM2_ESM.zip › EMBOR-2024-59287-SourceDataForFigure3C,3G,3H/3H/Blot_Input_anti-GFP-Marker.tif]

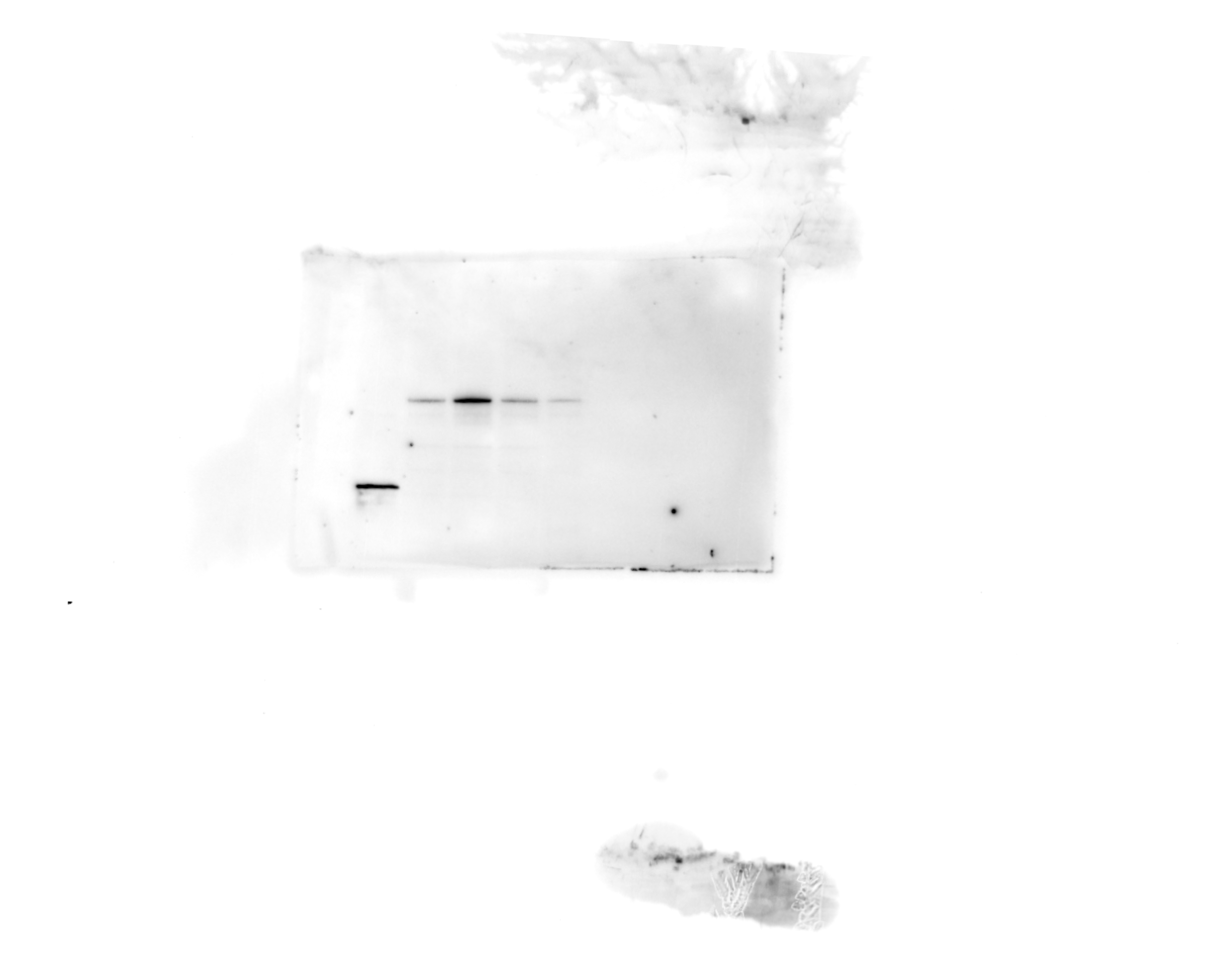

Supplement: Supplementary file 2 — Source data Fig. 3 [file 44319_2024_266_MOESM2_ESM.zip › EMBOR-2024-59287-SourceDataForFigure3C,3G,3H/3H/Blot_Input_anti-GFP.tif]

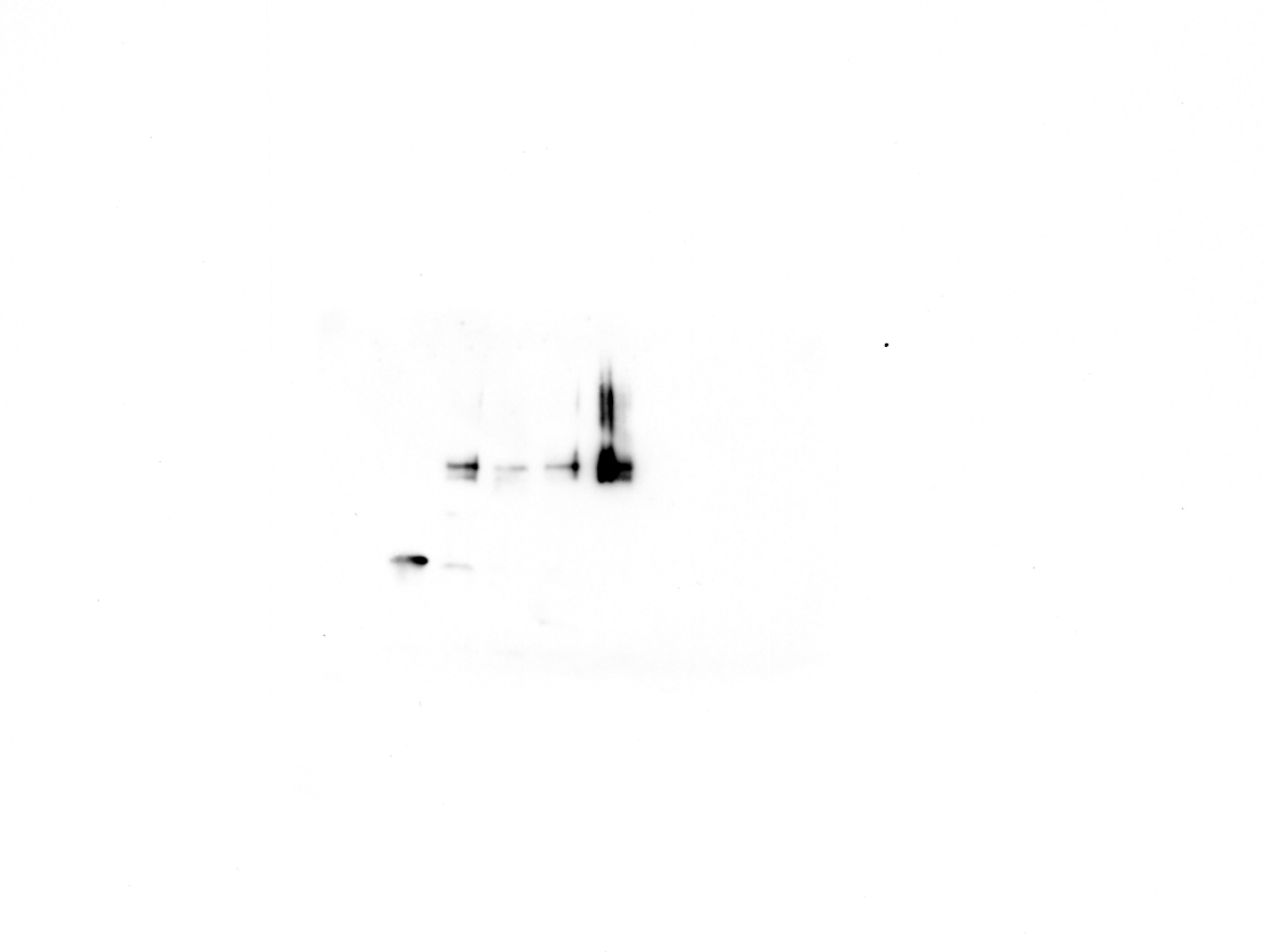

Supplement: Supplementary file 2 — Source data Fig. 3 [file 44319_2024_266_MOESM2_ESM.zip › EMBOR-2024-59287-SourceDataForFigure3C,3G,3H/3H/Blot_IP_Anti-GFP.tif]

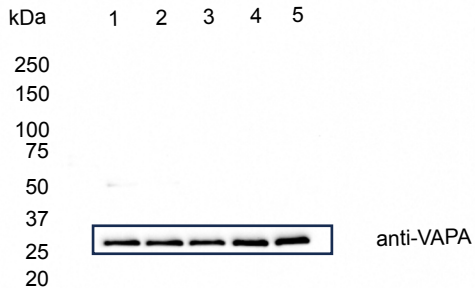

- 1 GFP
- 2 *CbEPF1*-wt-GFP
- 3 *CbEPF1*-F1mt-GFP
- 4 *CbEPF1*-F2mt-GFP
- 5 *CbEPF1*-F3mt-GFP

Supplement: Supplementary file 2 — Source data Fig. 3 [file 44319_2024_266_MOESM2_ESM.zip › EMBOR-2024-59287-SourceDataForFigure3C,3G,3H/3H/Blot_Input_anti-VAPA-Annotated.pdf]

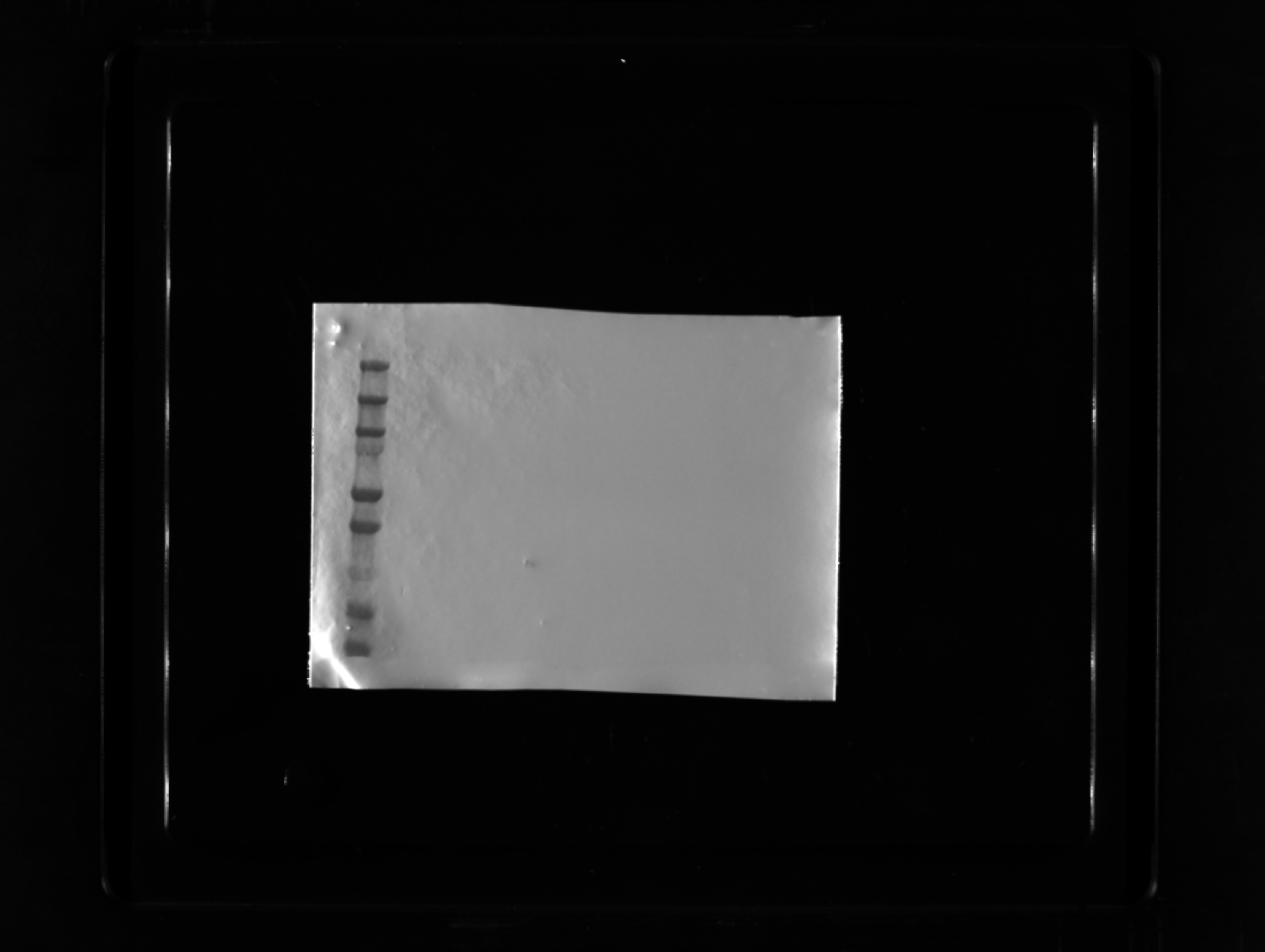

Supplement: Supplementary file 2 — Source data Fig. 3 [file 44319_2024_266_MOESM2_ESM.zip › EMBOR-2024-59287-SourceDataForFigure3C,3G,3H/3H/Blot_IP_Anti-GFP-Marker.tif]

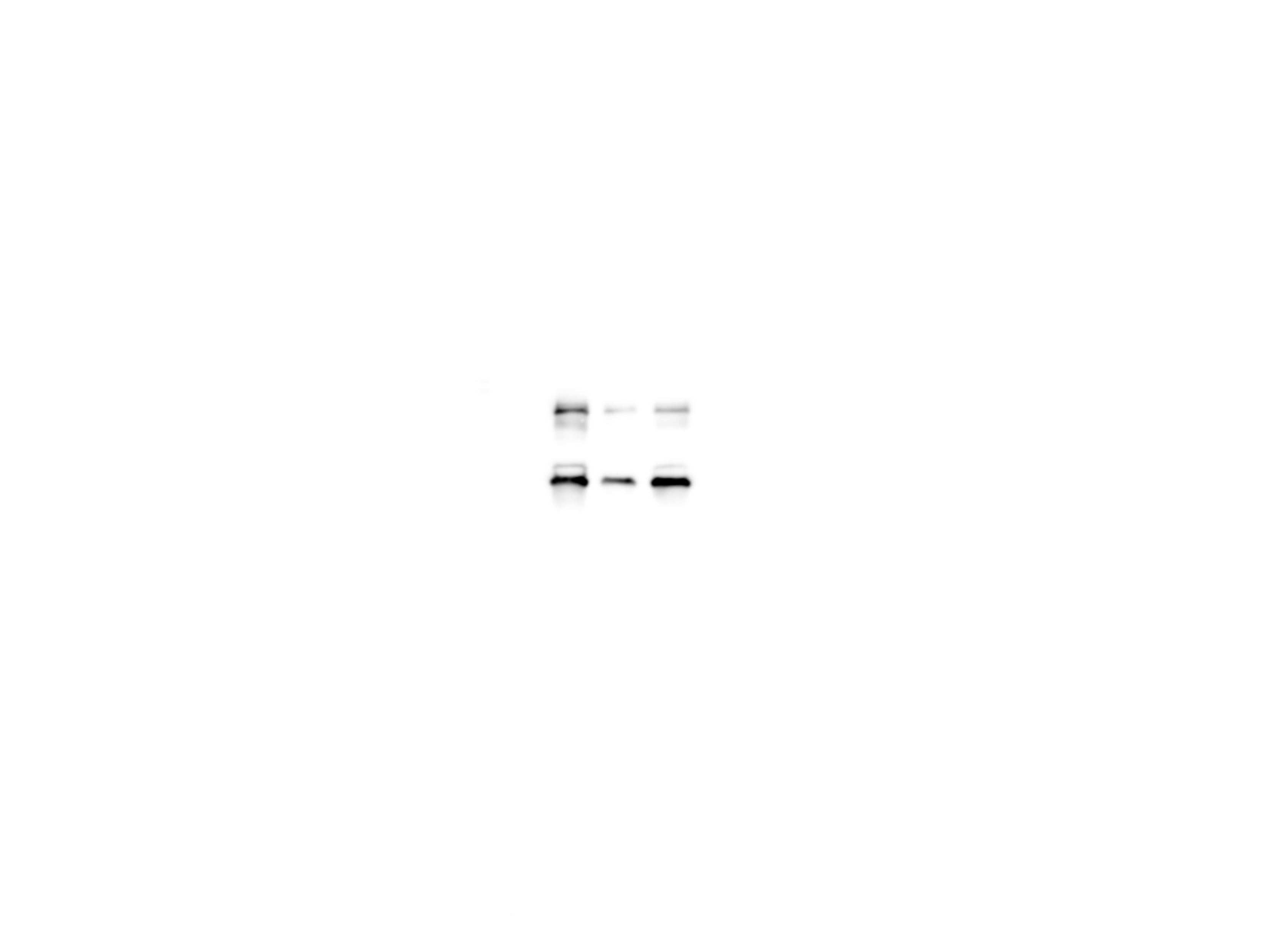

Supplement: Supplementary file 2 — Source data Fig. 3 [file 44319_2024_266_MOESM2_ESM.zip › EMBOR-2024-59287-SourceDataForFigure3C,3G,3H/3H/Blot_IP_anti-VAPA.tif]

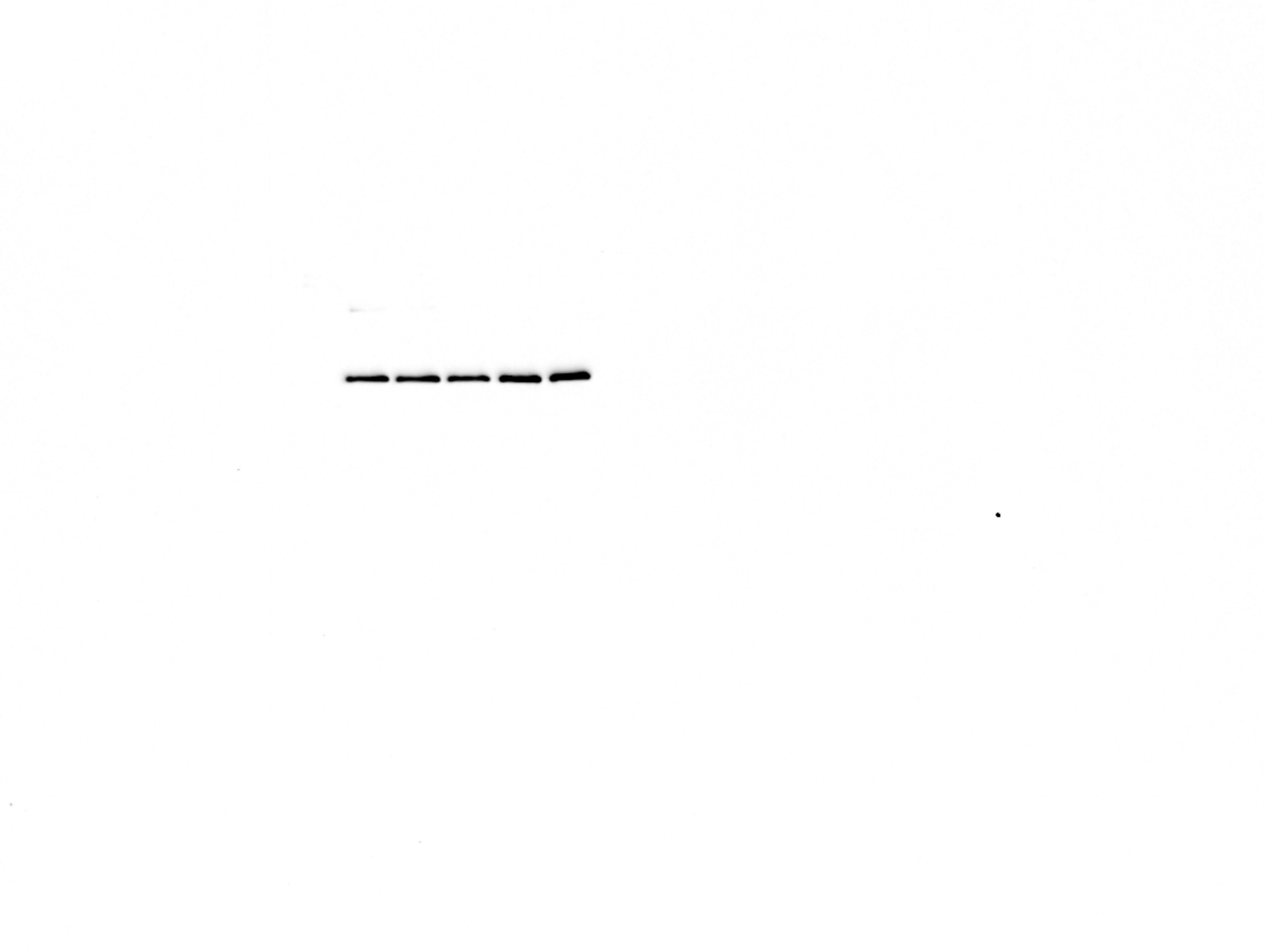

Supplement: Supplementary file 2 — Source data Fig. 3 [file 44319_2024_266_MOESM2_ESM.zip › EMBOR-2024-59287-SourceDataForFigure3C,3G,3H/3H/Blot_Input_anti-VAPA.tif]

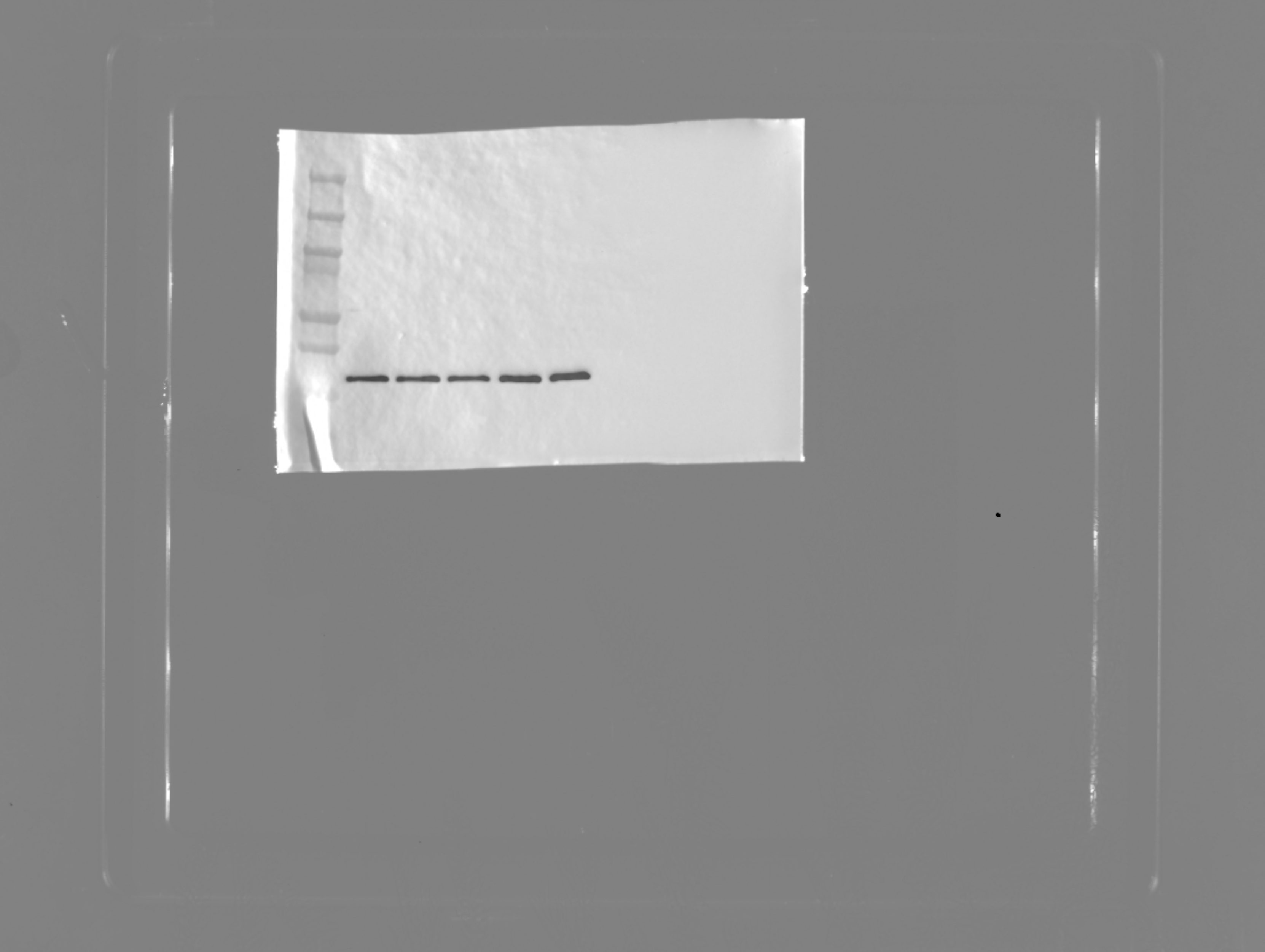

Supplement: Supplementary file 2 — Source data Fig. 3 [file 44319_2024_266_MOESM2_ESM.zip › EMBOR-2024-59287-SourceDataForFigure3C,3G,3H/3H/Blot_Input_anti-VAPA-Merged.tif]

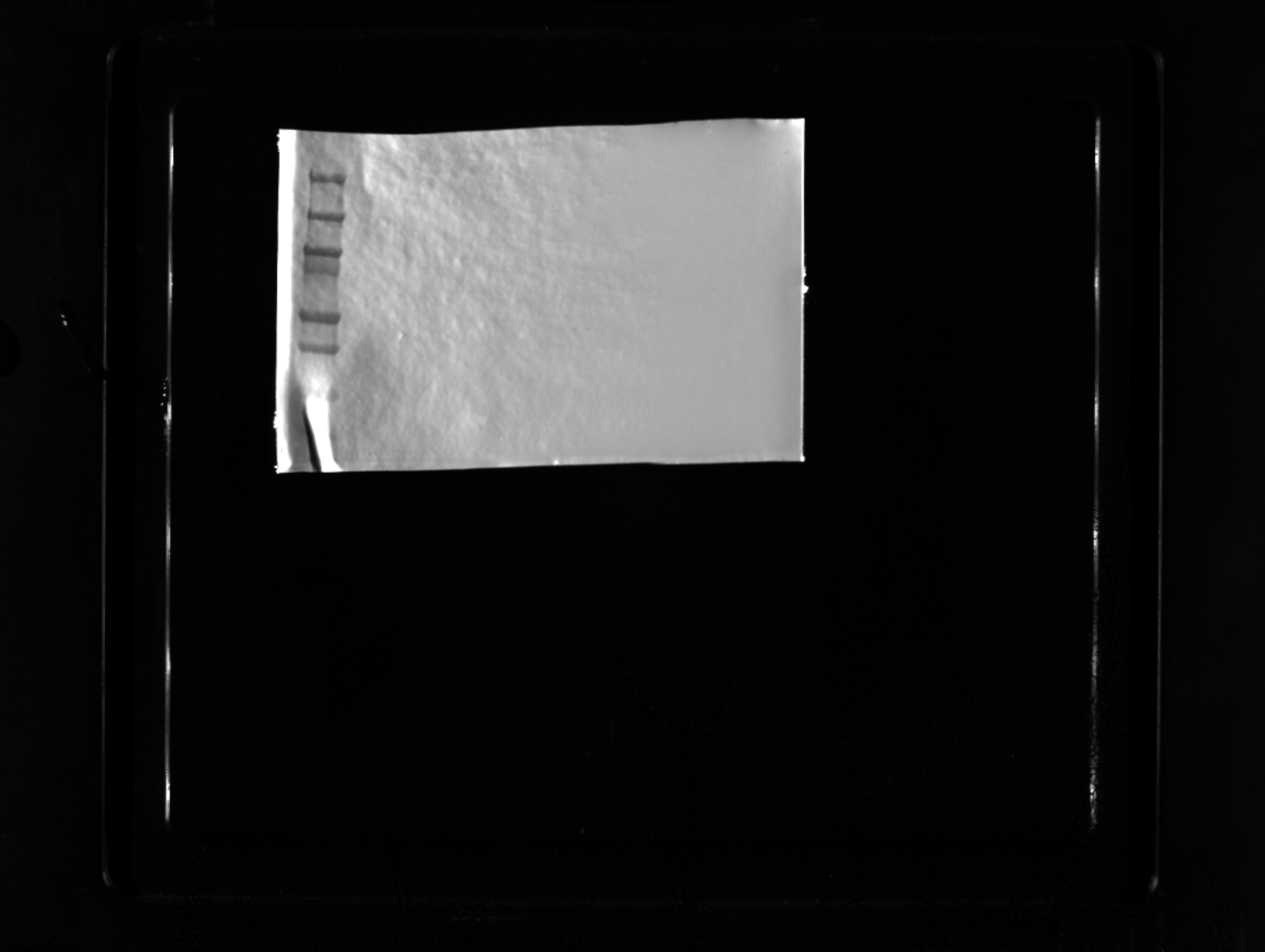

Supplement: Supplementary file 2 — Source data Fig. 3 [file 44319_2024_266_MOESM2_ESM.zip › EMBOR-2024-59287-SourceDataForFigure3C,3G,3H/3H/Blot_Input_anti-VAPA-Marker.tif]

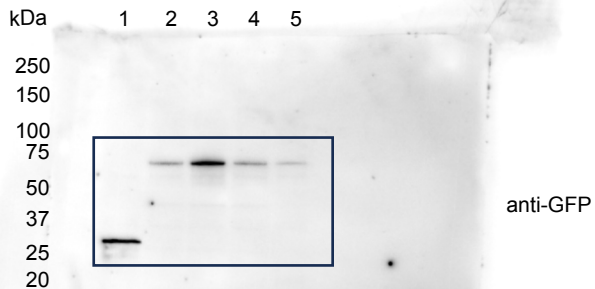

- 1 GFP  
2 *CbEPF1*-wt-GFP  
3 *CbEPF1*-F1mt-GFP  
4 *CbEPF1*-F2mt-GFP  
5 *CbEPF1*-F3mt-GFP

Supplement: Supplementary file 2 — Source data Fig. 3 [file 44319_2024_266_MOESM2_ESM.zip › EMBOR-2024-59287-SourceDataForFigure3C,3G,3H/3H/Blot_Input_anti-GFP-Annotated.pdf]

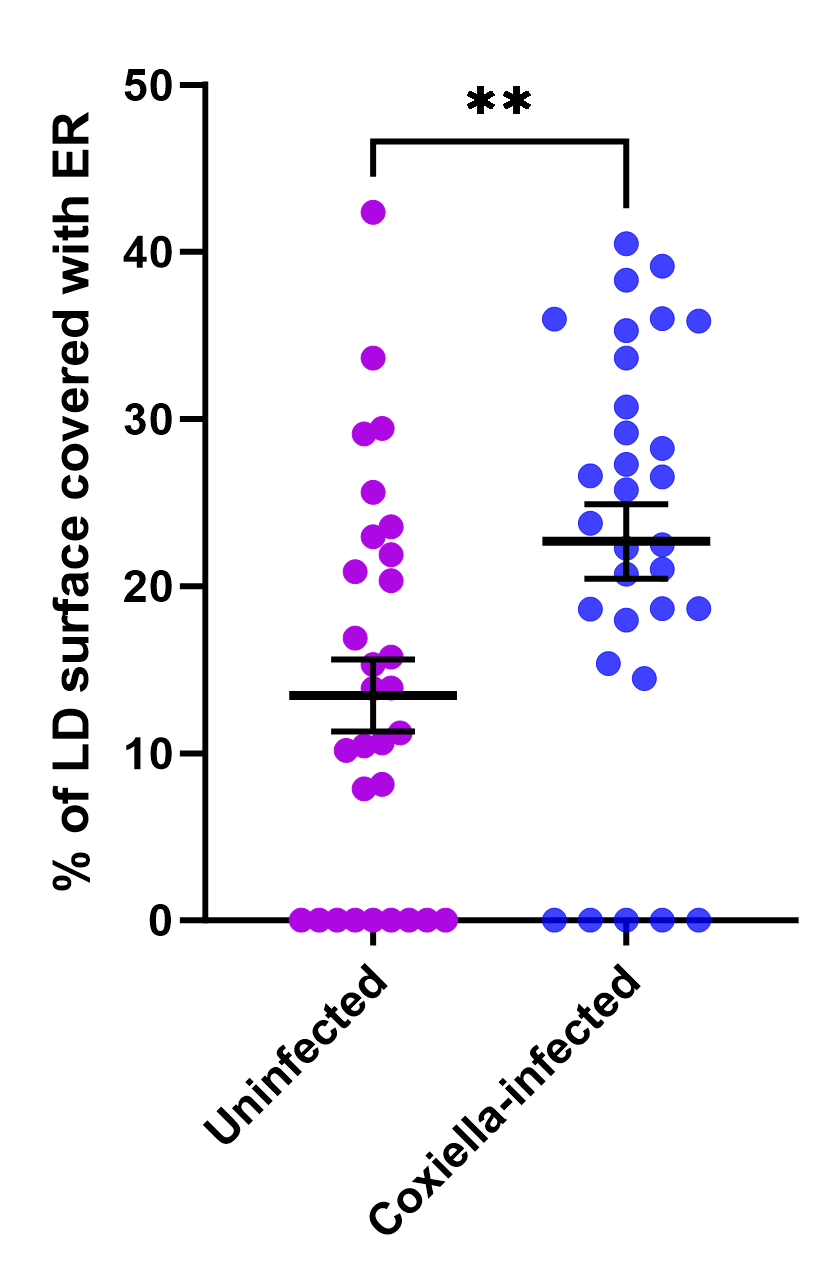

Supplement: Supplementary file 3 — Source data Fig. 5 [file 44319_2024_266_MOESM3_ESM.zip › EMBOR-2024-59287-SourceDataForFigure5B/5B/Percentage of LD perimeter covered by ER.tif]

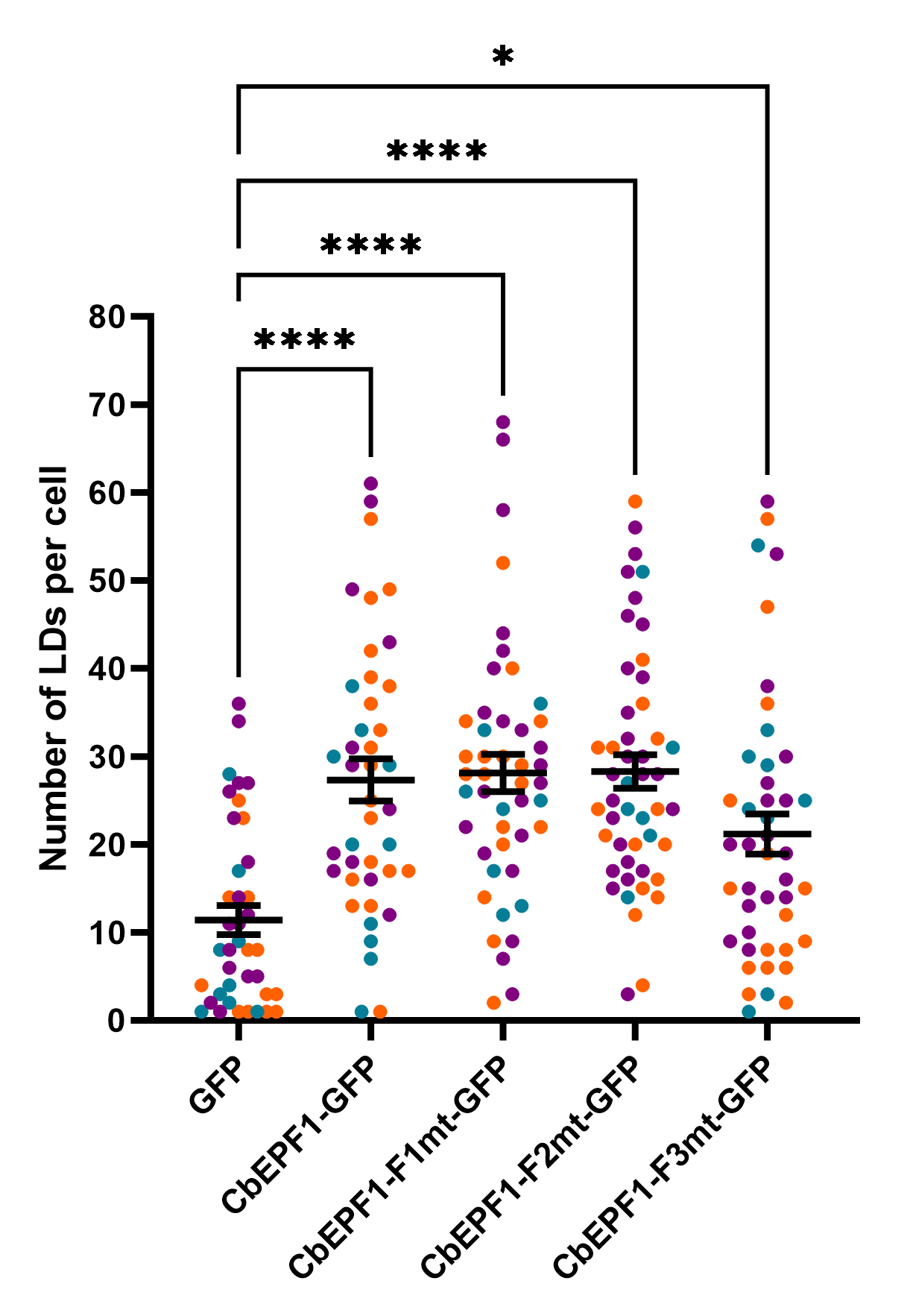

Supplement: Supplementary file 4 — Source data Fig. 7 [file 44319_2024_266_MOESM4_ESM.zip › EMBOR-2024-59287-SourceDataForFigure7A,7B/7A/EMBOR-2024-59287_NumericalData_SourceFigure_LDnumber.tif]

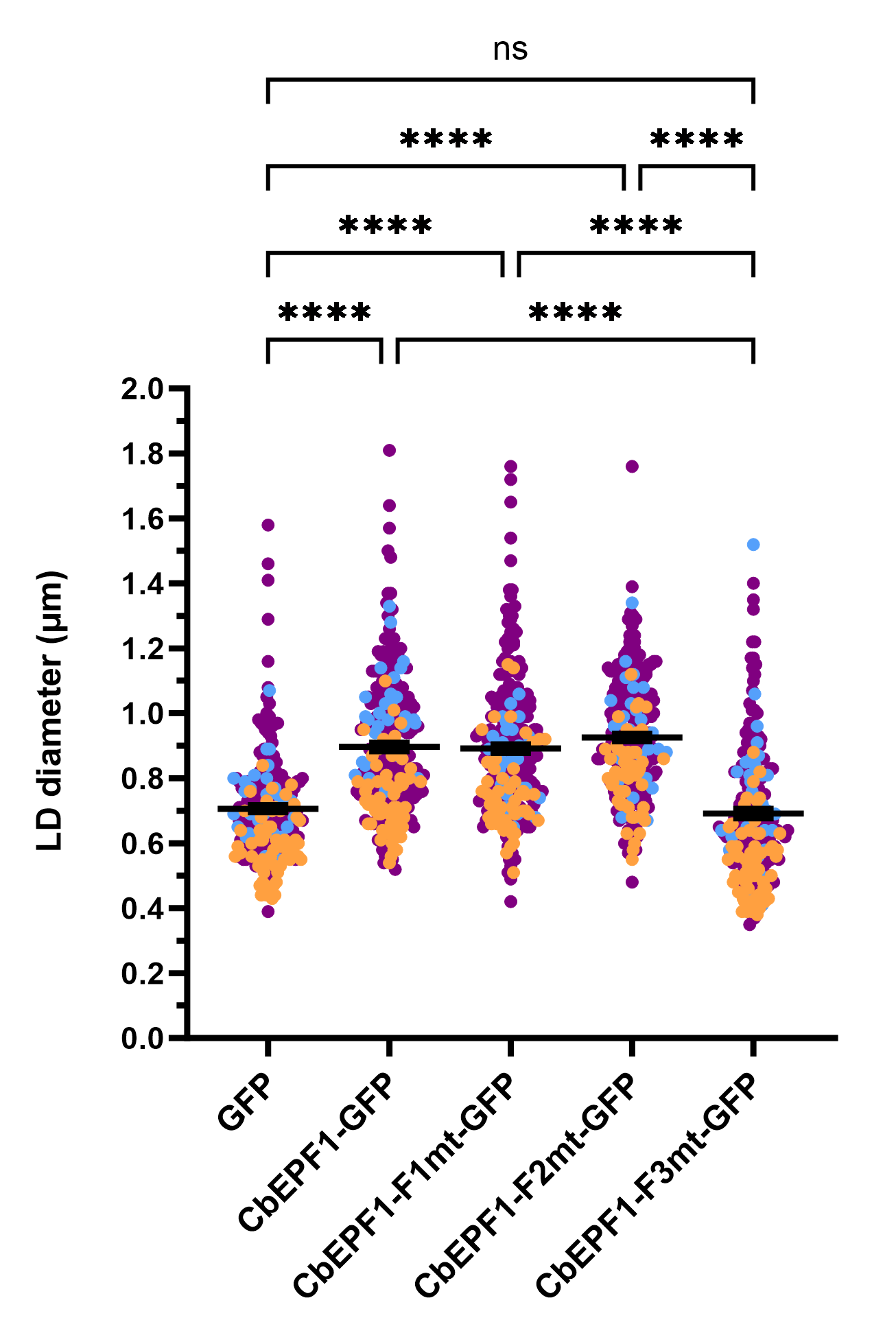

Supplement: Supplementary file 4 — Source data Fig. 7 [file 44319_2024_266_MOESM4_ESM.zip › EMBOR-2024-59287-SourceDataForFigure7A,7B/7B/EMBOR-2024-59287_NumericalData_Sourcefigure_LDsize.tif]
