# Supplementary material for: A novel bacterial effector protein mediates ER-LD membrane contacts to regulate host lipid droplets
Source: EMBO Rep. 2024 Sep 27;25(12):11. doi: 10.1038/s44319-024-00266-8 (PMC11624262; doi:10.1038/s44319-024-00266-8)
Supplement: Supplementary file 5 — Expanded View Figures [file 44319_2024_266_MOESM5_ESM.pdf]

Expanded View Figures

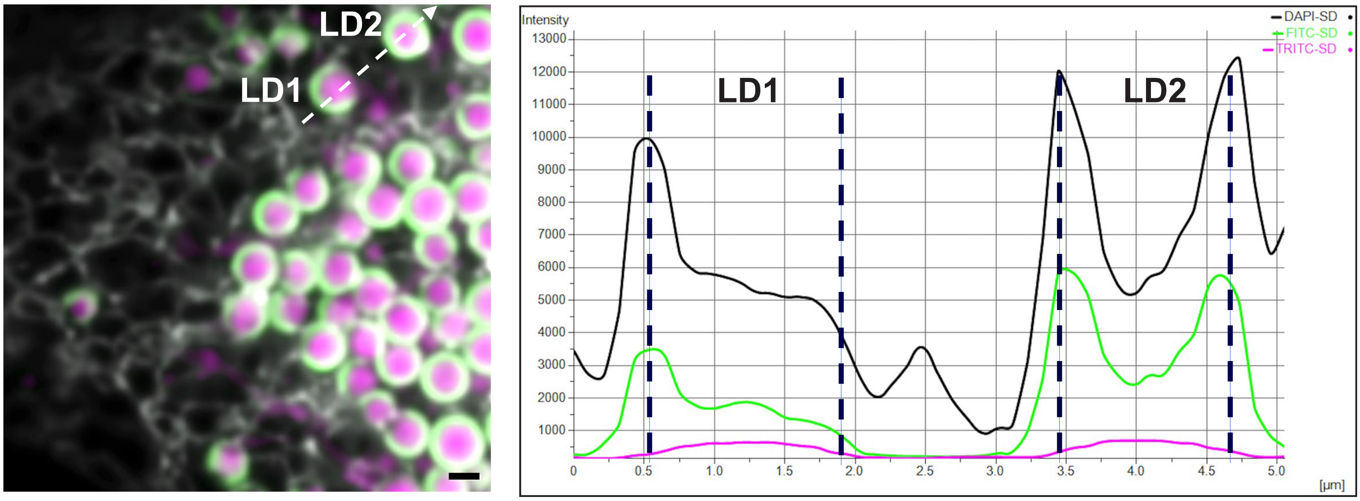

Figure EV1. *Cb*EPF1-GFP and BFP-KDEL localization along lipid droplets.

Intensity profiles of *Cb*EPF1-GFP and BFP-KDEL along the two lipid droplets (dotted lines). The shown image is from Fig. 2B bottom panel, which was reused for representation and intensity profile analysis. Scale bar on merged image: 1 µm. Intensity profiles: Black—BFP-KDEL, Green—*Cb*EPF1-GFP, and Magenta—LipidTOX red.

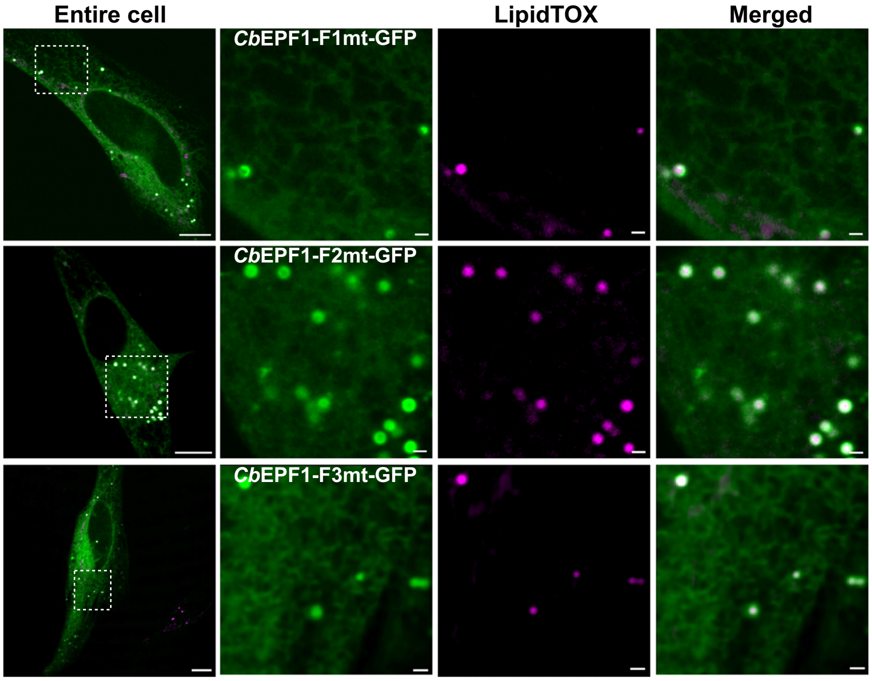

Figure EV2. Localization of *Cb*EPF1-FFAT mutant-GFP in HeLa cells.

The ectopically expressed *Cb*EPF1-F1mt-GFP or *Cb*EPF1-F2mt-GFP or *Cb*EPF1-F3mt-GFP localized to ER and LDs in HeLa cells. The *Cb*EPF1-FFAT mutant-GFP proteins showed a similar localization pattern to *Cb*EPF1-GFP (Fig. 1B). The LDs were labeled with LipidTOX-Red. Scale bars: 10 μm (overview) and 1 μm (magnified).

 © The Author(s)

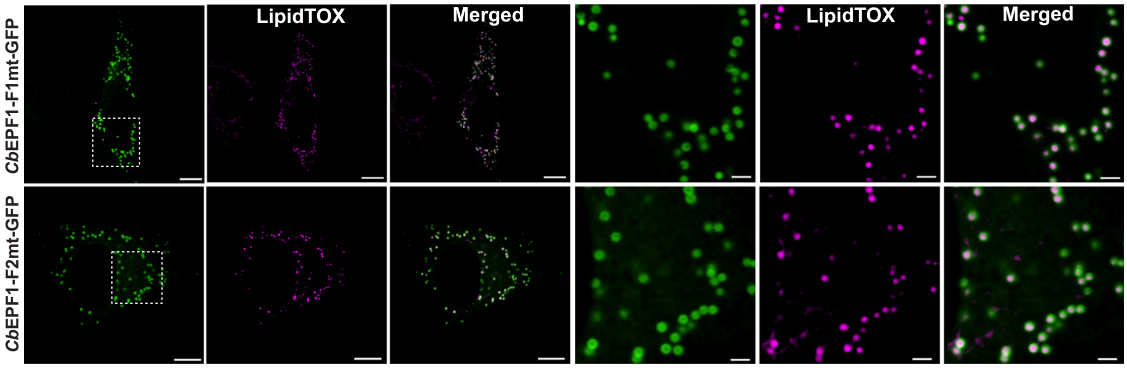

Figure EV3. Dispersion of LDs in cells expressing *Cb*EPF1-F1/F2 mutant-GFP.

Cells expressing *Cb*EPF1-F1mt-GFP or *Cb*EPF1-F2mt-GFP exhibit dispersed LDs in the HeLa cells. Scale bars: 10 μm (overview) and 1 μm (magnified).

© The Author(s)
